# Temporal evolution of beta bursts in the parkinsonian cortical and basal ganglia network

**DOI:** 10.1073/pnas.1819975116

**Published:** 2019-07-24

**Authors:** Hayriye Cagnan, Nicolas Mallet, Christian K. E. Moll, Alessandro Gulberti, Abbey B. Holt, Manfred Westphal, Christian Gerloff, Andreas K. Engel, Wolfgang Hamel, Peter J. Magill, Peter Brown, Andrew Sharott

**Affiliations:** ^a^Medical Research Council Brain Network Dynamics Unit, Department of Pharmacology, University of Oxford, OX1 3TH Oxford, United Kingdom;; ^b^Nuffield Department of Clinical Neurosciences, John Radcliffe Hospital, University of Oxford, Oxford OX3 9DU, United Kingdom;; ^c^Institut des Maladies Neurodégénératives, Universite de Bordeaux, 33076 Bordeaux, France;; ^d^CNRS UMR 5293, Institut des Maladies Neurodégénératives, 33076 Bordeaux, France;; ^e^Department of Neurophysiology and Pathophysiology, University Medical Center Hamburg-Eppendorf, 20246 Hamburg, Germany;; ^f^Department of Neurosurgery, University Medical Center Hamburg-Eppendorf, 20246 Hamburg, Germany;; ^g^Department of Neurology, University Medical Center Hamburg-Eppendorf, 20246 Hamburg, Germany;; ^h^Oxford Parkinson’s Disease Centre, University of Oxford, OX1 3QX Oxford, United Kingdom

**Keywords:** Parkinson’s disease, basal ganglia, beta oscillation, cortex

## Abstract

Beta frequency oscillations (15 to 35 Hz) in cortical and basal ganglia circuits become abnormally synchronized in Parkinson’s disease (PD). How excessive beta oscillations emerge in these circuits is unclear. We addressed this issue by defining the firing properties of basal ganglia neurons around the emergence of cortical beta bursts (β bursts), transient (50 to 350 ms) increases in the beta amplitude of cortical signals. In PD patients, the phase locking of background spiking activity in the subthalamic nucleus (STN) to frontal electroencephalograms preceded the onset and followed the temporal profile of cortical β bursts, with conditions of synchronization consistent within and across bursts. Neuronal ensemble recordings in multiple basal ganglia structures of parkinsonian rats revealed that these dynamics were recapitulated in STN, but also in external globus pallidus and striatum. The onset of consistent phase-locking conditions was preceded by abrupt phase slips between cortical and basal ganglia ensemble signals. Single-unit recordings demonstrated that ensemble-level properties of synchronization were not underlain by changes in firing rate but, rather, by the timing of action potentials in relation to cortical oscillation phase. Notably, the preferred angle of phase-locked action potential firing in each basal ganglia structure was shifted during burst initiation, then maintained stable phase relations during the burst. Subthalamic, pallidal, and striatal neurons engaged and disengaged with cortical β bursts to different extents and timings. The temporal evolution of cortical and basal ganglia synchronization is cell type-selective, which could be key for the generation/ maintenance of excessive beta oscillations in parkinsonism.

## Significance

Prevalence and temporal dynamics of transient oscillations in the beta frequency band (15 to 35 Hz), referred to as β bursts, are correlated with motor performance. Disturbance of these activities is a candidate mechanism for motor impairment in Parkinson’s disease (PD), where the excessively long bursts correlate with symptom severity and are reduced by pharmacological and surgical treatments. Here we describe the changes in action potential firing that take place across multiple nodes of the cortical and basal ganglia circuit as these transient oscillations evolve. These analyses provide fresh insights into the network dynamics of β bursts that can guide novel strategies to interfere with their generation and maintenance in PD.

Cortical oscillations in the beta frequency band (15 to 35 Hz) are thought to play a central role in cortical processing of information related to movement and cognition (1, 2). In Parkinson’s disease (PD), cortical beta oscillations become abnormally synchronized through their interaction with the dopamine-depleted basal ganglia network (3, 4). These pathological beta oscillations are suppressed when continuous high-frequency deep brain stimulation (DBS) is applied to either subthalamic nucleus (STN) or the internal segment of the globus pallidus (5–7), suggesting a strong dependence on a distributed subcortical network architecture for their generation and maintenance. A central goal in the field is to define the neuronal interactions through which abnormally strong and sustained beta oscillations emerge following dopamine depletion. Addressing these pathologically exaggerated activities may also give key insights as to how oscillations are transmitted in the healthy brain (1).

The spiking activity of neurons across the basal ganglia locks to specific phases of cortical oscillations in the parkinsonian brain (8–12). The high stability of these conditions of synchronization is likely determined by synaptic connectivity between different neuronal populations in the network, be it physiological, pathological, or compensatory. Elucidating the mechanisms of network synchronization is made harder by the large variance in baseline firing rates and presence/absence of autonomous firing in different basal ganglia cell types. Several hypotheses have been raised as to which network connections are necessary and sufficient for cortical oscillations to be abnormally propagated and/or amplified. These theories include 1) the excitatory–inhibitory coupling between the STN and the external segment of the globus pallidus (GPe) (13–15); 2) alteration of striatal output (12), possibly due to interneuron dysfunction (16–18); and 3) enhanced pallidostriatal positive feedback (19). These hypotheses have been based around the idea that oscillatory dynamics are relatively stable. However, recent studies have revealed that beta band activity in healthy (20) and parkinsonian basal ganglia local field potentials (LFPs) and frontal electroencephalograms (EEG) (21, 22) occurs in transient beta bursts (β bursts). It is currently unclear whether cortical and basal ganglia phase locking occurs predominantly during such bursts. If this is the case, the question of how beta oscillations are propagated and amplified can potentially be approached by identifying the neuronal interactions that occur before, during, and after these transient events. Such questions have assumed clinical significance due to the development of closed-loop stimulation approaches to DBS (23), as they have the potential to reveal the most effective way to disrupt the emergence of pathological oscillations.

Here we use multiple neuronal signals and analytical approaches in PD patients and dopamine-depleted rodents to demonstrate that cortical β bursts are associated with highly stable cortical and basal ganglia phase locking. These conditions of synchronization are established significantly earlier than the threshold commonly used to define the onset of β bursts using oscillation amplitude. Moreover, this initial period of synchronization is associated with the cell type-specific phase trajectories of basal ganglia spikes on the cortical beta oscillation. These findings have important implications for identifying the process through which neuronal oscillations propagate through different brain areas and for the design of closed-loop DBS.

## Results

The definition of a β burst varies extensively across different studies, in terms of both the threshold used to categorize an epoch of activity as a β burst, as well as the temporal dynamics of the identified burst (20–22, 24). In this study, the occurrence of a cortical β burst was inclusively defined as any period lasting >50 ms when the instantaneous beta amplitude of the Fz–Cz EEG (in the case of PD patients) or frontal electrocorticogram (ECoG, in parkinsonian rats) exceeded the 75th percentile of the amplitude calculated across the entire recording (21, 22). While frontal EEG signals may include contributions from subcortical sources, in this context they have been widely used as a read-out of cortical activity and provide similar information to ECoG (11, 25).

### PD Patients.

#### Corticosubthalamic phase locking follows the time course of EEG β-burst amplitude in PD patients.

**Fig. 1.**
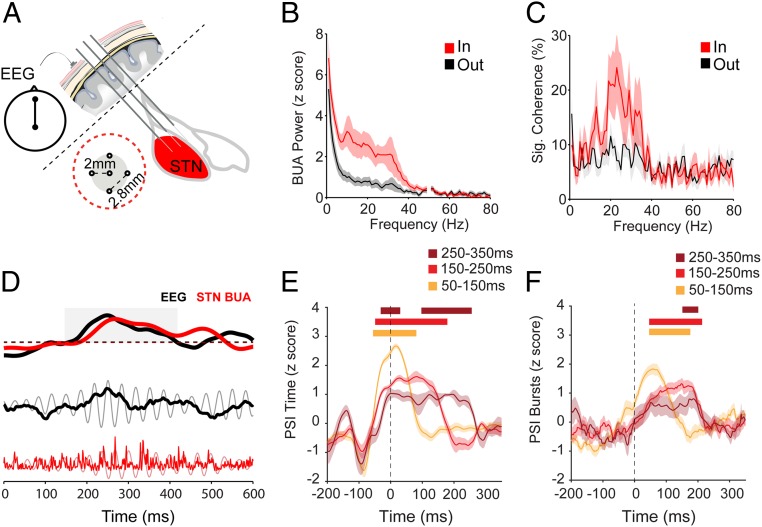
EEG β bursts are phase locked to spiking activity in the STN of PD patients. (*A*) BUA signals were recorded from 5 microelectrodes in the STN area together with EEG from the Fz–Cz position. As the microelectrodes traversed the STN, some electrodes were inside and some outside the structure. The layout of the 5 electrodes is shown within the red dotted circle. (*B*) Average BUA power spectrum from inside (red; *n* = 256 BUAs) and outside (gray; *n* = 222 BUAs) the STN, highlighting that enhanced rhythmic activity in the beta band (15 to 35 Hz) is observed inside the STN. (*C*) The proportion of BUA signals that are coherent with the EEG is selectively increased in the beta frequency range when electrodes were inside but not outside of the STN (number of recordings as in *B*). (*D*) An exemplary EEG β burst (black) from subject 1 (right hemisphere), together with simultaneously recorded STN-BUA (red). Onset of a cortical β burst was defined as the time point that the instantaneous beta amplitude exceeded the 75th percentile of the amplitude of the whole recording and remained elevated for at least 50 ms. Dashed line indicates the amplitude threshold for determining the onset of the EEG β burst. The beta amplitude of both the EEG and STN BUA simultaneously crosses the burst threshold and remains above it for around 200 ms (gray box). This increase in amplitude is visible in the raw (black) and filtered (gray) EEG signal. Bursts of activity in the STN BUA (raw, red; beta filtered, pink) are clearly aligned to the trough of the EEG beta oscillation during the burst. (*E* and *F*) Phase locking in time (*E*) and across bursts (*F*) around the onset of EEG β bursts (time 0; dotted line) between EEG_β_ and subthalamic BUA_β_ for different cortical β-burst durations (hemispheres = 13, patients = 7, *n* = 18 recordings; see color key for burst duration; see *SI Appendix*, Fig. S1, for schematic explanation of analysis methods). Significance for each burst length was determined using a cluster-based analysis (*Methods*) which tested the difference in the modulation of PSI over time in the burst-aligned data to that from randomly selected data that had no relationship to burst onset. Significant increases with respect to baseline are indicated with horizontal bars, color matched to the burst duration. *B* and *C* show mean and SEM across hemispheres. In *E* and *F*, shaded regions indicate the SEM across all recordings.

β bursts have been extensively reported in the STN LFPs of PD patients, but it is unclear to what extent they reflect STN unit activity. To address this issue, we utilized the background unit activity (BUA) signal recorded in and around the STN of PD patients undergoing intraoperative functional mapping for subsequent DBS therapy. The BUA signal is a continuous time series (like EEG/ECoG) and provides insight into the synchronous spike discharges of local neuronal ensembles (12). BUA recorded from the STN of PD patients exhibited enhanced rhythmic activity at beta frequencies (Fig. 1 *A* and *B*). Moreover, subthalamic BUA was strongly coherent with the EEG in the beta frequency band (Fig. 1*C*). Beta oscillations and significant coherence with EEG were confined to BUA signals within the physiologically defined boundary of the STN (Fig. 1 *B* and *C*). Having established that synchronous beta oscillations were present, we used these signals to address the question of whether the phase locking of subthalamic neuronal activity to the EEG followed the time course of EEG β bursts. To this end, after filtering EEG and BUA in the beta frequency band (EEG_β_ and STN-BUA_β_) we computed phase locking, as measured by the phase synchrony index (PSI) of the Hilbert transform-derived phase of these signals, around the onset of the EEG β bursts (hemispheres = 13, patients = 7, *n* = 18 recordings). Significant modulation of the time course of all PSI analyses was assessed using a cluster-based test to compare the burst-aligned data with data that were randomly selected with no relation to burst onset (see *Methods* for details). PSI in time, which measures the strength of phase locking over short epochs (50 ms) across each individual burst (*SI Appendix*, Fig. S1*A*), was elevated around the β-burst onset, and the duration of locking increased with longer burst durations (Fig. 1*E*). Importantly, the onset of phase locking (in time) began on average −115, −114, and −35 ms relative to the onset of short (50 to 150 ms), medium (150 to 250 ms), and long (250 to 350 ms) bursts, respectively, suggesting that increased corticosubthalamic synchronization actually precedes the amplitude-based burst threshold. PSI across bursts, which measures the consistency of the phase relationship between bursts at each individual time point (*SI Appendix*, Fig. S1*B*), began on average −80, −74, and −79 ms for short, medium, and long bursts relative to the EEG β-burst threshold (Fig. 1*F*). The time course of PSI across bursts followed that of the amplitude less closely than that of PSI in time (Fig. 1*F*). These findings demonstrate that in PD patients, corticosubthalamic synchronization precedes the onset and follows the time course of EEG β bursts, and the conditions of this transient synchronization are consistent across bursts.

### 6-OHDA–Lesioned Rats.

Electrophysiological recordings in the basal ganglia of PD patients are mostly limited to the STN. To define how other areas of the basal ganglia network are modulated by cortical β bursts, we used multichannel silicon electrodes (or probes) to record unit activities in the GPe, striatum (Str), and/or STN of parkinsonian 6-OHDA–lesioned rats; frontal ECoGs were simultaneously recorded with these basal ganglia activities (9, 12). As for the EEG in the patient recordings, the Hilbert transform-derived phase and amplitude were extracted from the beta-filtered ECoG (ECoG_β_ phase and amplitude; see *Methods* for details of filtering). *SI Appendix*, Fig. S2, shows that ECoG β bursts of different lengths had broadly equivalent characteristics across these datasets (*SI Appendix*).

#### ECoG β bursts are associated with increased cortical and basal ganglia phase locking and local oscillations in the basal ganglia.

**Fig. 2.**
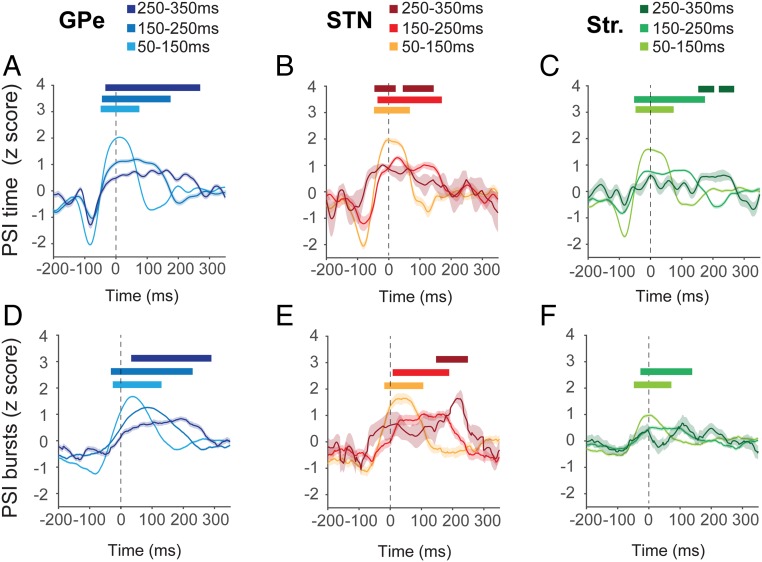
Cortical and basal ganglia phase locking at the onset of ECoG β bursts. (*A*–*C*) PSI, computed in time, between ECoG_β_ and each BUA_β_ in 3 basal ganglia structures aligned to the start of the ECoG β burst (time 0; dotted line). Points plotted indicate the beginning of the 50-ms-long moving window. (*D*–*F*) PSI, computed across bursts, between ECoG_β_ and basal ganglia BUA_β_ aligned to the start of the ECoG β burst. In each case, bursts have been separated into 3 different durations (see color keys). Both analyses used the same number of BUA signals (GPe = 366, STN = 22, Str = 209). Significance for each burst length was determined using a cluster-based analysis (*Methods*) which tested the difference in the modulation of PSI over time in the burst-aligned data to that from randomly selected data that had no relationship to burst onset. Significant increases with respect to baseline are indicated with horizontal bars, color matched to the burst duration. (Shaded regions indicate the SEM across all recordings made from a given structure.)

As shown in PD patients**,** STN BUA_β_ in 6-OHDA–lesioned rats exhibited increases in phase locking to ECoG_β_ slightly before and during the ECoG β burst (Fig. 2). This transient synchronization extended to the GPe and Str BUA_β_ phase_,_ suggesting that it is a network-wide phenomenon (Fig. 2 *A* and *C*). Across all structures and burst lengths, increases in both PSI over time and PSI across bursts preceded the ECoG β-burst threshold (Fig. 2 and *SI Appendix*, Tables S1 and S2). The same analyses, aligned to burst offset, demonstrated that the time at which ECoG_β_ amplitude dropped below the β-burst threshold correlated with the loss of phase locking with the basal ganglia BUA_β_ signals (*SI Appendix*, Fig. S3). These results demonstrate that transient increases in cortical and basal ganglia phase locking occur in individual bursts, with stable parameters of synchronization between bursts across the BG. To test this further, we triggered raw, unfiltered BUA signals at the burst threshold, after adjusting the trigger to the nearest peak of the beta phase, whereby changes in phase alignment or frequency of the signals would weaken the timing of activity in relation to time and/or phase. In each structure, the alignment of the raw BUA with the ECoG β phase around the time of the β burst was consistent enough, across bursts and recordings, for the beta oscillation to emerge simply from summing the unfiltered activity around a single time point (*SI Appendix*, Fig. S4). As in the analysis of PSI, this phase-driven modulation appeared to start 1 or more cycles before the burst threshold in all structures (*SI Appendix*, Fig. S4).

To establish whether this increase in cortical and basal ganglia phase locking was associated with a concurrent increase in local beta oscillations in the basal ganglia structures, the evolution of BUA_β_ amplitude (i.e., the magnitude of the Hilbert Transform) in STN, GPe, and Str was computed in relation to ECoG β-burst onset for the 3 burst durations (*SI Appendix*, Fig. S5). In GPe, longer ECoG β bursts were clearly associated with longer periods of increased BUA_β_ amplitude following ECoG β-burst onset (*SI Appendix*, Fig. S5). In Str and STN, BUA_β_ amplitude generally increased following the β-burst threshold, but the relationship with burst duration was less clear (*SI Appendix*, Fig. S5). Overall, the time course of ECoG_β_ amplitude during β bursts was associated with parallel modulation of cortical and basal ganglia phase locking and basal ganglia oscillation across the network.

##### Conditions for cortical and basal ganglia phase locking are established before the ECoG β burst.

**Fig. 3.**
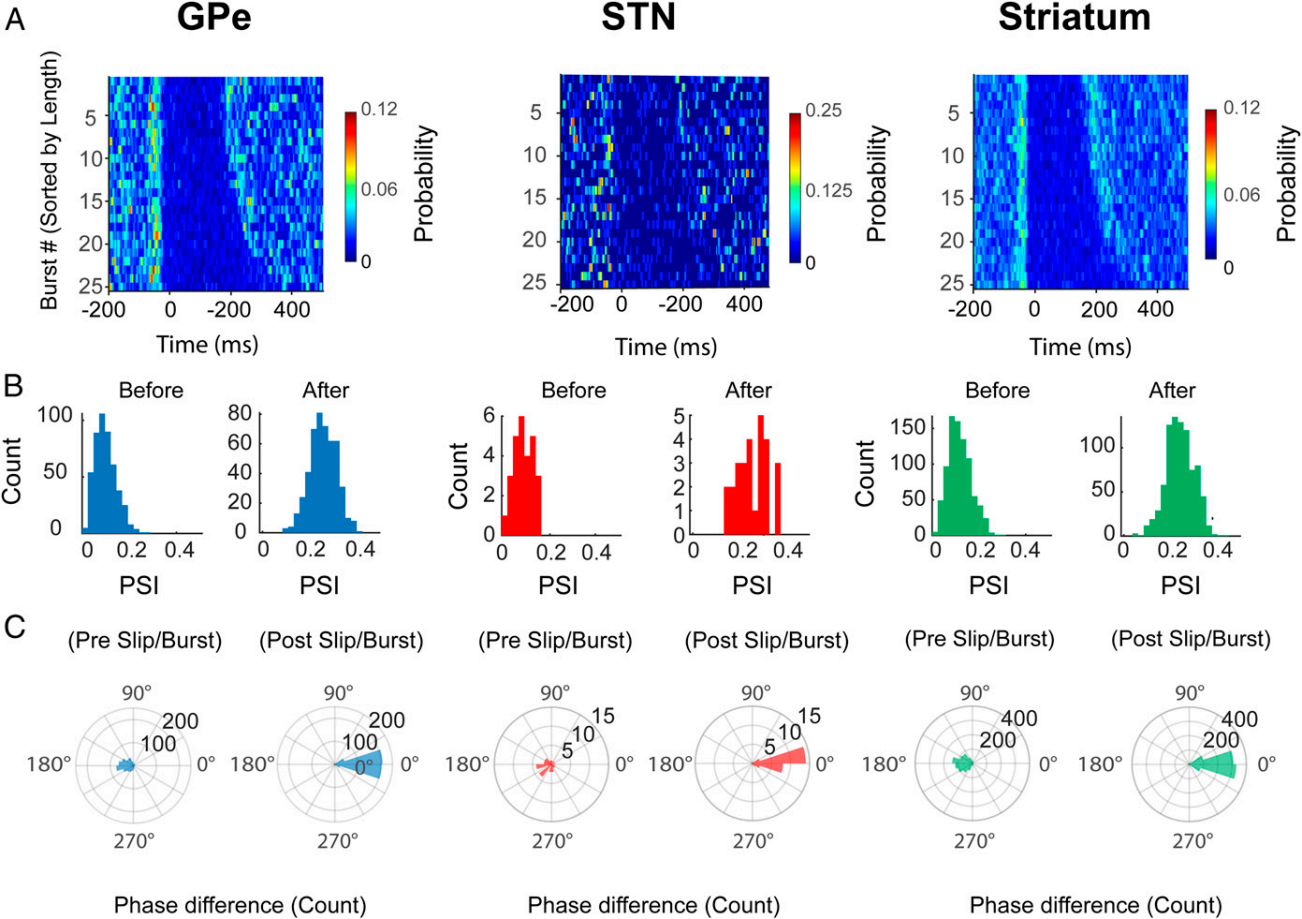
Phase slips indicate that conditions for cortical and basal ganglia phase locking have been established before the onset of ECoG β bursts. (*A*) Average phase slips observed during the 25 longest ECoG β bursts in each recording. The phase alignment between the ECoG_β_ and BUA_β_ altered before the cortical burst onset (time = 0), defined according to the threshold crossing. (*B*) Phase alignment between the ECoG and the basal ganglia BUA_β_, before a phase slip, was not consistent across different epochs, highlighted by low PSI values. After phase slip, phase alignment was around twice as consistent across different trials. (*C*) Phase alignment immediately before a phase slip differed from the phase alignment observed during the ECoG β burst. However, immediately after a phase slip, the phase alignment between signals was the same as the phase alignment observed during the ECoG β burst (i.e., 0° difference between the phase of alignment after the slip and during the burst). (In *B* and *C*, GPe = 366, STN = 22, and Str = 209 BUA signals.)

The analyses above demonstrate that phase locking begins significantly before the ECoG burst threshold and that the conditions of synchronization (i.e., the phase difference between the signals) are consistent between bursts. Next we tested whether it was possible to detect the point at which these stable phase conditions are established. To address this question, we calculated the probability of phase slips, defined here as abrupt changes in the phase difference of the ECoG_β_ and basal ganglia BUA_β_ phase, in relation to the ECoG β-burst onset (Fig. 3). The average probability of a phase slip in the 200 ms before β-burst onset was greater than the average probability of a phase slip occurring in the following 200 ms (paired *t* test *P* < 0.001 for all regions). To determine whether these phase slips were predictive of the onset of stable synchronization, we compared the conditions of phase alignment immediately before and after the slip to those that occurred within the β burst itself. We observed that the angle of the phase difference between the ECoG_β_ and BG BUA_β_ immediately before a phase slip was inconsistent across β-burst epochs, as revealed by low PSI values, in all structures (Fig. 3*B*). Immediately after a phase slip, the PSI values more than doubled (Fig. 3*B*). Crucially, we found that the precise angle of the phase difference occurring immediately after the phase slip was the same as that within the burst itself (Fig. 3*C*, as indicated by a difference of 0°). In contrast, there was a weak relationship between the preburst phase difference and that within the burst, with a slight tendency for the opposite alignment (Fig. 3*C*, as indicated by a difference of 180°). These findings were repeated across all BG structures and were consistent with an increase in the variance in BUA_β_ frequency before the burst threshold, which was significant in Str and GPe (*SI Appendix*, Fig. S6). The same analysis of the differentiated unwrapped Hilbert phase of each of the ECoG_β_ and BUA_β_ individually showed that with the exception of the striatal BUA, phase slips were also more likely to occur in the 200 ms before the burst threshold in all structures (*SI Appendix*, Fig. S7). While the alignment of phase slips was more pronounced in cortex, any comparison must take into account the caveat that the BUA signals are likely produced by more local ensembles of neurons. In addition, repeating these analyses following the removal of trials where the instantaneous frequency had a negative value (i.e., where the oscillatory cycle completely breaks down) did not significantly change the results shown in Fig. 3 (*SI Appendix*, Fig. S8), suggesting that complete breakdown of the oscillatory cycle did not underlie these findings. Overall, these analyses highlight that at the level of local ensembles of neurons, abrupt changes in the alignment of BG spiking activity and cortical beta oscillations precede transiently stable conditions in cortical and basal ganglia phase locking.

##### What is the relationship of transient increases in cortical and basal ganglia phase locking to the firing rate and pattern of basal ganglia neurons?

BUA provides an effective way of measuring recruitment of ensemble activity within a given structure to cortical activity but could mask important differences in specific populations of neurons within those structures (9, 26). In addition, BUA signals do not allow the delineation of changes in firing rate and recruitment of neurons to the oscillation. To address these issues, we performed complementary analyses using sorted action potentials of single neurons and distinguished between the 2 major populations of neurons in the GPe, based on well-established criteria (9). GP-TI neurons fire most around the inactive phase of the cortical slow oscillation and have been shown to correspond to prototypic GPe neurons that project to the STN. In contrast, GP-TA neurons fire most around active phase of the cortical slow oscillations and correspond to arkypallidal neurons that project only to Str (26, 27). The mean phase angles calculated over the entire recording are shown in *SI Appendix*, Fig. S9, and firing rates of these neurons are described in *SI Appendix*, Table S3. The subset of striatal neurons used here (*n* = 104), which were significantly locked to cortical beta, were likely to be indirect pathway spiny projection neurons (12).

**Fig. 4.**
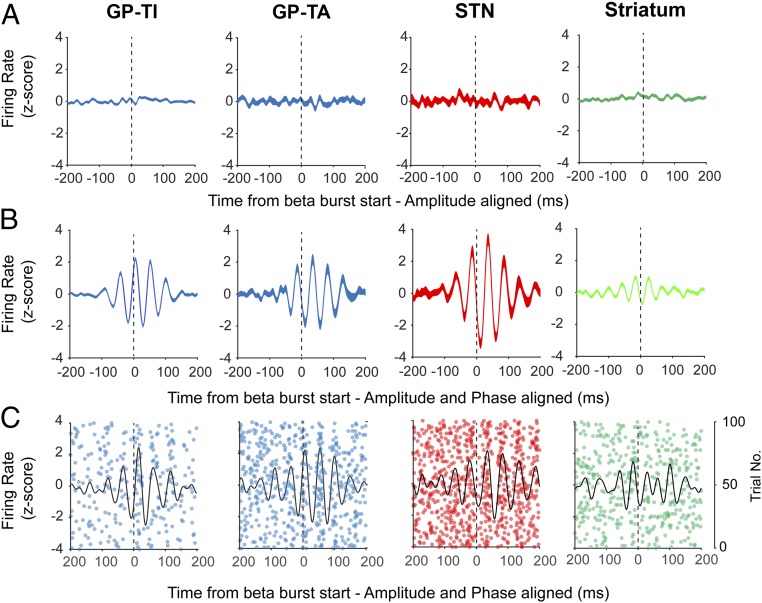
Basal ganglia units transiently phase lock to ECoG β bursts without modulation of firing rate. (*A*) Mean z-score firing rate of the burst onset-triggered (time 0, 75th percentile of amplitude) single units in each structure, with no adjustment for the cortical beta phase. Firing rate is not altered during the burst. (*B*) The same analysis but the burst onset trigger is shifted to the nearest peak in the cortical beta phase, aligning the phase of each burst around the trigger. Firing rate is significantly modulated by the phase alignment to the cortical oscillation and offset with respect to time 0. Numbers of units were the same for both analyses (GP-TI = 179, GP-TA = 40, STN = 18, Str = 104). (*C*) Representative examples of unit activity from the Str, GP-TA, GP-TI, and STN. Each subpanel shows spike timings derived from a single unit, across individual cortical bursts. Black lines indicate the corresponding averaged activity for that unit realigned to the time point that cortical beta amplitude crossed median levels.

To evaluate how the rate and pattern of action potentials in the basal ganglia were modulated by cortical β bursts we first computed triggered averages, similar to the widely used spike-triggered average, using the burst onset points throughout the recording as the triggers (*SI Appendix*, Fig. S10*A*). As with spike-triggered averages, this simple technique can reveal both oscillatory and nonoscillatory modulations of instantaneous firing rate on multiple time scales. We first triggered unit activity by the burst onset points defined only by amplitude. This method did not result in any modulation of firing rate in any of the BG populations at any time scale (Fig. 4*A*), demonstrating that overall rate of action potentials is not altered on the time scale of whole bursts (see *SI Appendix*, Fig. S10*A*, for example of how the opposite result could occur). When the burst triggers were adjusted to nearest oscillation peak, however, there were clear oscillatory modulations in the firing pattern of each population at beta frequency in line with the cortical β-burst time course (Fig. 4*B*). This demonstrates that modulation of the burst-triggered raw BUA (*SI Appendix*, Fig. S4) is likely underpinned by the timing of action potentials from single neurons. In line with this level of explanation, individual GP-TI, GP-TA, Str, and STN units exhibited clear oscillatory firing at the onset of ECoG β bursts (Fig. 4*C*). For individual GP and STN units, it is noteworthy that each β burst (i.e., trial on the raster plot) could evoke a highly consistent pattern of oscillatory firing for over 200 ms (Fig. 4*C*). Simulations of this analysis, under the assumption that the cortical amplitude reflects the probability that a unit will fire at a specific phase of the same oscillation, suggest that this method is resistant to changes in frequency, burst length, and firing rate within the range of variance present in the real data (*SI Appendix*, Fig. S11).

#### Temporal evolution of ECoG β bursts is associated with cell type-selective phase-locking trajectories of the basal ganglia neurons.

**Fig. 5.**
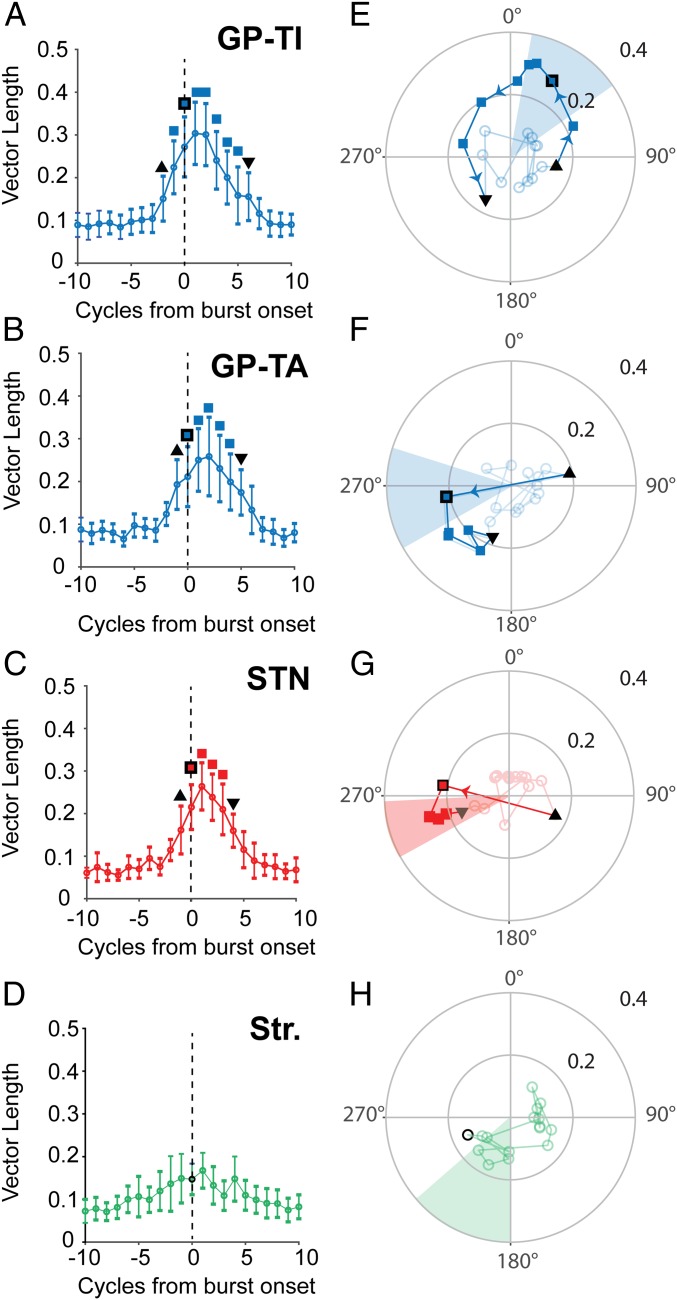
Temporal evolution of cortical and basal ganglia phase locking conditions during cortical β bursts. (*A*–*D*) Mean vector length with 1 STD (error bars) of the phase-locking action potentials to ECoG beta phase in the 10 oscillation cycles on either side of that containing the ECoG β-burst onset (cycle 0; dotted line) for the spikes of GP-TI (*A*; *n* = 151), GP-TA (*B*; *n* = 31), STN (*C*; *n* = 19), and striatal (*D*; *n* = 20) neurons. Symbols above data points indicate significant increases in vector length compared with any of the previous cycles (Kruskal–Wallis ANOVA with post hoc Dunn–Cidek tests). Black triangle, first significant increase in vector length; colored square with black outline, cycle containing burst threshold; inverted black triangle, last cycle with significantly increased vector length; colored squares; all other cycles with significantly increased vector length. The vector lengths of GP-TI, GP-TA, and STN neurons (*A*–*C*) were significantly different between cycles (Kruskal–Wallis ANOVA, *P* < 0.002), and post hoc tests showed that these differences occurred around the burst threshold. The vector length of striatal neurons (*D*) was significantly modulated by cycle position (Kruskal–Wallis ANOVA, *P* = 0.0012), but no individual cycle was significantly different to those occurring previously. (*E*–*H*) Preferred phase angle of spikes of each neuron type in each cycle. Each symbol shows the average vector length (distance from center) displayed in *A*–*D*, together with the corresponding preferred phase angle (position on circle) of spikes in each beta cycle. Faded circles show cycles with no significant increase in vector length. The remaining symbols are matched to the points on *A*–*D* so that progression in phase can be compared with that in vector length in relation to the burst threshold. Lines between the symbols indicating the cycle-to-cycle progression. The shaded areas show 1 STD around the mean β-phase, calculated across whole recordings, for each population. Significant changes in angle between consecutive preferred phases with significant vector length are denoted by the colored arrows (Watson–Williams test, *P* < 0.01).

Finally, we aimed to identify the precise temporal profiles and conditions of synchronization of action potentials recorded from specific populations of BG neurons as the amplitude of the ECoG β burst evolved. To this end, we divided each ECoG β burst into individual cycles around that containing the burst threshold (cycle 0). By using the β phases of all action potentials fired by a single neuron within a given cycle, across bursts, we could calculate cycle-by-cycle circular statistics in relation to burst onset (*SI Appendix*, Fig. S10*B*, and Fig. 5). In each neuronal population, the vector length across cycles was significantly modulated by the position of cycle in relation to the burst threshold (Kruskal–Wallis ANOVA, *P* < 0.002). GP-TI neurons (*n* = 151) displayed a significant increase in vector length 2 cycles before the burst onset (Fig. 5*A*), with around 20 and 40% of neurons significantly phase locking in the prethreshold cycles, respectively (*SI Appendix*, Fig. S12*A*). Notably, GP-TA neurons (*n* = 31) followed a similar pattern, but phase locking started after and ended before that of the GP-TI neurons (Fig. 5*B*). STN neurons (*n* = 19) also showed an increase in vector length 1 cycle before burst onset (Fig. 5*C*) but disengaged 1 cycle earlier than both types of GPe neuron (Fig. 5 *A–C*). For striatal neurons (*n* = 20), although vector length was significantly modulated by cycle position (Kruskal–Wallis ANOVA, *P* = 0.002), no individual cycle was significantly different from those preceding it (Fig. 5*D*). Despite this, around 20% of striatal neurons were significantly locked in the cycles surrounding the burst threshold, and this proportion decreased to 0 around the edges of the burst (*SI Appendix*, Fig. S12*A*). Cycle-by-cycle analysis confirmed that none of these neuron types displayed modulation of firing rate in relation to the burst onset (*SI Appendix*, Fig. S13). The spikes of GPe and STN neurons could phase lock to several consecutive cycles of the burst, suggesting oscillatory firing, whereas striatal neurons mostly locked to 1 or 2 cycles (*SI Appendix*, Fig. S12*B*).

Previous studies have provided detailed information regarding the mean ECoG beta phase (mean β phase) of spikes in each of these BG neuronal populations (9, 12, 26). However, our analysis of phase slips (Fig. 3) suggests that the alignment of spiking to the ECoG moves in phase to achieve the stable conditions of synchronization that occur during bursts. We hypothesized that phase slips before and stable conditions of synchronization during bursts would be reflected in the phase at which spikes locked to the ECoG β phase as the amplitude of the burst evolved. This proved to be particularly prominent for the spikes of GP-TI neurons, the preferred β phase of which moved around 3/4 of the oscillation cycle over the rise and fall of the ECoG β amplitude, settling for only a few cycles at their mean phase during the maximum amplitude of the burst (Fig. 5*E*). In contrast, the mean phase of GP-TA and STN spikes shifted dramatically between the cycles before and containing the burst threshold but then stayed at or close to their mean phase angles for the remaining cycles with increased vector length (Fig. 5 *F* and *G*). Despite their burst-related increase in vector length not reaching significance, the angle of locking of striatal action potentials still showed distinct structure, moving to the mean phase before and during the burst (Fig. 5*H*). When viewed together, the preburst phase locking angles of GPe and STN spikes were all at the descending phase of the oscillation, before moving to the mean β phases at point of burst onset (*SI Appendix*, Fig. S14). Notably, the phase trajectory of GP-TI spikes meant that they were within 1/4 of a cycle of those of GP-TA, STN, and striatal neurons immediately before phase-locking magnitude across all neurons dropped back to baseline (*SI Appendix*, Fig. S14). The analyses of striatal neurons (Fig. 5 *G* and *H*) was likely underpowered due to their low firing rate and tendency to lock to only 1 to 2 consecutive cycles (*SI Appendix*, Fig. S10*B*), which meant that few neurons accumulated enough spikes in each cycle to calculate circular statistics. To address this, for each population we generated resampled data where 500 spikes were randomly selected across the population for each cycle, and this was repeated 100 times. This allowed us to pool data from a larger number of neurons (i.e., spikes could be taken from neurons that did not have >20 spikes on each cycle). This analysis suggested that while smaller in magnitude than the other populations, the vector length of striatal spikes did significantly increase, with spikes firing at a consistent phase angle at or close to their mean β phase over the same time course as GP-TI neurons (*SI Appendix*, Fig. S15).

These findings suggest that temporal evolution of synchronization and oscillation in basal ganglia neuronal ensembles during cortical β bursts could be underlain by the precise phase relationships of specific populations of neurons to cortical beta oscillations and to each other.

## Discussion

Beta oscillations in the cortex and basal ganglia occur in transient bursts of increased amplitude (20, 21, 24, 28). Here we show the time course of cortical and basal ganglia synchronization follows that of the cortical beta amplitude at the level of ensembles and single neurons and that the conditions of synchronization are consistent for each burst. The initiation, peak, and offset of cortical bursts is associated with cell type-selective trajectories of spiking in relation to the cortical phase. Thus, the generation, maintenance, and termination of network synchronization is likely underpinned by the synaptic interactions between these connected groups of neurons that evolve over the time scale of the burst.

### Transient Cortical and Basal Ganglia Phase Locking Occurs Independently of Firing Rate.

Oscillations and synchronization have become increasingly prominent in explaining the pathophysiology of the parkinsonian brain; however, there is considerable evidence that changes in firing rate across the basal ganglia play also an important role (29). Distinguishing changes in the timing of activity from those of firing rate/excitability is therefore crucial for delineating the neuronal activity that leads to parkinsonian symptoms and for the role of these activities in healthy processing. In the context of PD, the Str is particularly important in this regard as dopamine-based changes in excitability of spiny projection neurons are central to rate-based models of pathophysiology (30). The striatal neurons that contributed most heavily to the striatal BUA here were likely to be GPe projecting, indirect pathway spiny projection neurons (iSPNs), which become hyperactive following dopamine depletion (12, 31). Importantly, the magnitude of hyperactivity of iSPNs is positively correlated with the strength of their phase locking to cortical beta oscillations (12), suggesting that modulation of excitability is necessary for iSPNs to propagate cortical rhythms. Here we found that striatal neurons increased their cortical phase locking during β bursts, without any change in firing rate over the same time scale. Together, these findings suggest different time scales for pathophysiological changes in rate/excitability and phase locking and that the former is not the result of the latter. Despite this, the very low firing rate of SPNs under heathy conditions means that the tonic, background increase in iSPN excitability following dopamine depletion could still be crucial for these neurons to propagate cortical oscillations downstream (12). In contrast, in STN and GPe, much of the firing is driven by intrinsic currents (32). As a result, small perturbations to timing and synchronization of spiking can have a bigger impact on output than changes in excitability (28). Thus, for these populations it is less surprising that profound modulations in timing of action potentials can occur independently of rate changes, and this is consistent with evidence that modulation of oscillation, rather than rate, best predicts the effectiveness of therapy (7). Overall, our results suggest that changes in the excitability of basal ganglia neurons do not interact with beta oscillations and synchrony on the subsecond time scale.

### Identifying Cortical and Basal Ganglia Interactions at the Onset of β Bursts.

Since the discovery that beta oscillations are abnormally synchronized in PD patients, a central question in the field has been how these activities are generated and propagated within cortical, basal ganglia and thalamic circuits. In patients, there is significant evidence that cortical oscillations lead those in basal ganglia (11, 33–36). For this reason, we used the EEG or ECoG amplitude as our reference point for the timing of other activities. However, given the looping nature of cortical and basal ganglia circuits, it seems likely that even if the cortical oscillations provide an initial reference frame, the subsequent time course of cortical amplitude is the result of interactions across the network that evolve over time. We provide an approach to this issue that utilizes the transient nature of β bursts to examine network events that occur around their onset.

Phase locking of each BG population started before the ECoG burst threshold. We do not interpret this as the BG driving the cortical burst but rather that there is a period before the burst threshold where the spikes align to a specific phase of the cortical oscillation. This early alignment of cortical and basal ganglia activity could provide the initial conditions through which a beta oscillation can be amplified and propagated across the network. Thus, it is noteworthy that phase locking in GP-TI neurons started 1 cycle before that of STN neurons. For the burst initiation, this result is at odds with experiments in MPTP-lesioned primates suggesting that beta is propagated through the corticosubthalamic hyperdirect pathway (14, 37). Rather, it suggests that in rats, cortical oscillations initially influence GPe neurons via iSPNs, their other main source of input. In line with this observation, highly oscillatory corticosubthalamic oscillations fail to pattern GPe unless dopamine is depleted and/or striatal neurons are also coupled to the oscillation (38, 39). Our recent finding that identified iSPNs lock to cortical beta oscillations provides further evidence for a possible role for striatal outputs (12). Here analysis of striatal neurons did not provide conclusive evidence to support or refute this hypothesis. Despite recording hundreds of striatal neurons, their low firing rate meant that only a fraction could be analyzed using the cycle-by-cycle method (i.e., having enough spikes in each cycle to calculate a reliable vector length). Indeed, consistent with their low firing rate, they tended to lock to only 1 or 2 cycles of the β burst. This low rate and sparse nature of striatal firing, combined with the huge volume and number of neurons in the structure, means that even higher-density recordings may be necessary to fully evaluate their role. Whatever the role of striatum, our results are consistent with a key role for GPe in network synchronization (26, 27). Even if STN is influenced by the indirect before the hyperdirect pathway, however, it could still be the case that convergence of the 2 pathways on to STN and GPe neurons could promote the highest levels of synchronization and oscillation amplitude. It is also noteworthy that GP-TI neurons were also a cycle ahead of GP-TA neurons (which correspond to striatal-projecting arkypallidal neurons), demonstrating that neuronal populations can have their own temporal dynamics with respect to afferent input and/or network activities even within a basal ganglia nucleus.

At the population level, the onset of phase locking could be detected as a slip in the phase relationship between the ECoG and BUA, after which the conditions of synchronization remained constant until burst offset. It seems likely that these population level events were underlain by the shifts in the preferred angle of phase locking that we observed in the spiking of all of the BG populations close to the burst threshold. In the case of STN and GP-TA neurons, the phase of spikes shifted abruptly over 1 cycle, before settling at the mean phase for those populations. In contrast, the preferred phase of GP-TI spikes moved steadily along the oscillation as the amplitude evolved across cycles, settling only briefly at the peak of the burst amplitude. Notably, the progression of the GP-TI phase meant that it eventually reached the mean phase of the other populations, at which point phase locking to cortex was lost. The axons of GP-TI neurons are in the unique position to inhibit all of the other populations simultaneously (26). Thus, the movement of GP-TI spikes between antiphase and in-phase relationships with their targets could determine whether network was more or less likely to enter and/or maintain a synchronous oscillation over time. If this is the case, an important question raised by our findings is how and why the spikes of GP-TI neurons continue to change their preferred cortical phase over time, while the other populations were relatively stable. The answer to this question might reveal how and why some β bursts last longer than others.

### Implications for the Development of Closed-Loop Stimulation.

In addition to being of importance to uncovering pathophysiology mechanisms, these findings have important implications for the development of closed-loop stimulation in PD and other brain disorders. One of the most promising closed-loop approaches is adaptive DBS, where high-frequency STN stimulation is delivered when instantaneous beta amplitude crosses an amplitude (i.e., burst) threshold (23). Here we demonstrate that the commonly used burst threshold is a good predictor of the time point at which basal ganglia populations have reach phase-locking stability at their mean preferred phases, providing physiological rationale for this biomarker. Our findings suggest that it may be possible to go a step further than adaptive DBS and interact with neuronal events that precede these stable dynamics and disrupt their generation. A tractable approach to achieving this would be to use phase, rather than amplitude, as a biomarker to control the timing of stimulation as phase alignment consistently preceded amplitude changes across different signals and analyses. Phase-dependent stimulation using peripheral signals can reduce the amplitude of tremor by up to half (40, 41). While beta oscillations are more transient than tremor and require a central signal to track phase, we and others have already provided proof-of principle evidence that this approach can also suppress beta oscillations in the parkinsonian brain (7, 42). Stimulation of 1 of the BG structures at a specific cortical beta phase could prevent BG spikes from entering the phase-locking conditions associated with network β bursts, either by preventing firing at some phases and/or driving spikes at others. Given the ubiquitous nature of transient oscillations in the cortex and basal ganglia, the ability to selectively alter the events underlying network oscillations could provide a powerful scientific and therapeutic tool.

## Methods

### Intraoperative Recordings.

#### Patient details and clinical scores.

The present study was conducted in agreement with the Code of Ethics of the World Medical Association (Declaration of Helsinki, 1967) and received local ethics approval (Ethik-Kommission Ärztekammer Hamburg). Written informed consent was given by all patients who participated in this study. Cortical EEGs and microelectrode recordings from the STN were recorded from 7 patients (4 female, 3 male; age 67 ± 3 y) with advanced idiopathic PD with a disease duration of 17 ± 8 y, during bilateral implantation of DBS electrodes in the STN, guided by microelectrode mapping. Clinical details are summarized in *SI Appendix*, Table S4.

#### Microelectrode recordings.

Microelectrode recordings were acquired from 5 electrodes using a MicroGuide interoperative recording system (Alpha-Omega). While the central electrode aimed at the theoretical target (*SI Appendix*, *Supplementary Materials*), the remaining 4 electrodes were placed 2 mm around it. Signals were amplified (×20,000), sampled at 24 kHz, and filtered between 300 and 6,000 Hz. Electrode positions were classified as being in the STN using several previously described criteria: a well-defined elevation in the background activity (43, 44), together with presence of action potential discharge patterns with tonic irregular, oscillatory, or burst-firing features. These features allowed for a distinction to be made from the neighboring substantia nigra pars reticulata thalamus/zona incerta neurons. Cortical EEG signals were simultaneously recorded and sampled at 3,005 Hz, amplified, and filtered (×4,000; bandpass, 0 to 400 Hz); see Moll et al. (45) and Sharott et al. (11) for details. EEG signals were recorded approximately from the Fz and Cz using Ag/AgCl cup electrodes filled with conductive gel (Nicolet Biomedical). In this study, the Fz was referenced to the Cz.

### Recordings from Parkinsonian Rats.

#### In vivo electrophysiological recording with multielectrode arrays.

Simultaneous extracellular recordings of unit activity and LFPs were made from numerous sites in the dorsal Str, GPe, and STN of urethane-anesthetized 6-OHDA–lesioned rats. Striatal recordings were made without any other electrodes in the brain (*n* = 7 animals, *n* = 89 recordings). In some cases, STN and GPe recordings were made simultaneously (*n* = 9 animals, *n* = 25 recordings), and in others, GPe was recorded alone (*n* = 9 animals, *n* = 19 recordings). All signals were recorded together with coincident epidural ECoG, which was recorded with a 1-mm-diameter screw above the frontal, motor cortex. Further details on 6-hydroxydopamine lesions and silicon probe recordings can be found in *SI Appendix*, *Supplementary Materials*.

### Data Analysis.

Recordings were analyzed using custom-written software in MATLAB. Throughout the paper, the beta frequency band is defined as 15 to 35 Hz. From the recordings described above, in this study we have made use of the following types of signals.

#### BUA.

In experimental parkinsonism, BUA was derived from raw probe recordings by high-pass filtering the recordings at 300 Hz using a third-order Butterworth filter. We then identified spiking activity by setting a threshold at mean + 4 SD of the recording, removed a 4-ms segment around each epoch crossing this threshold, and replaced it with part of the recording that did not contain any spiking activity. Following removal of spiking activity, the data were rectified and low-pass filtered at 300 Hz using a third-order Butterworth filter. For STN microelectrode recordings from PD patients, the signal was recorded using a band-pass filter (300 to 6,000 Hz). The other processing steps were identical to processing of BUA in experimental parkinsonism. BUA activity in the beta frequency band (BUA_β_) was derived by filtering (second-order Butterworth filter) the BUA activity ±5 Hz around the frequency with highest coherence with Fz–Cz EEG/frontal ECoG within the beta frequency band.

#### Single-unit activity.

Single-unit activity was extracted from the band-pass filtered (500 to 6,000 Hz) wide-band probe signals according to the following criteria: 1) signal/noise ratio of >2.5 and 2) spike sorting using methods such as template matching, principal component analysis, and supervised clustering (Spike2 Cambridge Electronic Design Limited). Units were classified as single units if a distinct refractory period in the interspike interval histograms could be identified.

#### Spectral analysis of EEG and STN BUA in PD patients.

EEG and STN BUA signals were downsampled to 512 Hz (MATLAB function “resample”). BUA power and EEG-BUA coherence were evaluated with fast Fourier transform-based methods using the MATLAB toolbox Neurospec, as described previously (46). BUA power and coherence between the EEG and each coincident BUA signal were calculated with a FFT size of 512, giving a frequency resolution of 1 Hz. Each BUA power spectrum was normalized using the mean SD across high-frequency spectral values (100 to 150 Hz) to convert each 1- to 80-Hz power value into a z score. The normalized power spectra were then averaged for each hemisphere. Fig. 1*B* shows the mean and SEM of these z-scored spectra for each hemisphere. For each coherence spectra, significance in 1- to 80-Hz bins was evaluated using 95% confidence limits based on the number of segments used and was independent of frequency (46). For each hemisphere/STN, we calculated the percentage of significantly coherent EEG/BUA pairs for each frequency. Fig. 1*C* shows the mean and SEM of the percentage of significant bins in a given frequency across hemispheres. BUA signals were assigned as being inside or outside the STN based on the physiologically defined borders of the structure for that electrode (*SI Appendix*, *Methods*).

#### β-Burst definition.

β-burst onset was derived from the Fz–Cz EEG in PD patients and from frontal ECoG in 6-OHDA–lesioned rats. An epoch was labeled as a β burst if the instantaneous beta amplitude of these signals exceeded the 75th percentile of amplitudes over the entire recording for at least 1 beta cycle (i.e., 50 ms). β-burst onset and offset (i.e., first and last points above the 75th percentile threshold) were adjusted to the nearest 0° of the cortical beta phase (within ±30 ms of the threshold crossing) unless otherwise stated. Bursts with a duration of 50 ms or less were excluded from further analysis due to ambiguity associated with the nature of transients that last less than 1 beta cycle (47). Cortical beta phase was derived from the angle of the Hilbert transform.

#### Phase locking and PSI.

For all PSI analyses, basal ganglia BUA and EEG/ECoG were filtered ±5 Hz around the peak BUA-EEG/ECoG coherence frequency (BUA_β_ and EEG_β_/ECoG_β)_. Instantaneous phase was derived from the Hilbert transform of the BUA_β_ and EEG_β_/ECoG_β_. Phase alignment (ϕ_j_) was computed by subtracting the instantaneous phase of the BUA_β_ from that of the EEG/ECoG_β_. Phase alignment values were corrected to be within ± π by adding 2π when phase alignment was less than −π or subtracting 2π when phase alignment was greater than π. PSI in time was computed by dividing the burst period into overlapping 50-ms epoch and deriving the PSI for each epoch using the following formula: $\left|\left(\sum_{\text{j}=1}^{\text{j}=\text{N}}{\text{expe}}^{-\text{i}\emptyset_{\text{j}}}\right)\right|/\text{N}$, where N was the number of samples corresponding to 50 ms (see *SI Appendix*, Fig. S1*A*, for schematic description). PSI across bursts was computed by deriving PSI across β bursts using the following formula: $\left|\left(\sum_{\text{j}=1}^{\text{j}=\text{N}}{\text{expe}}^{-\text{i}\emptyset_{\text{j}}}\right)\right|/\text{N}$, where N was the total number of ECoG/EEG β bursts of a certain duration (see *SI Appendix*, Fig. S1*B*, for schematic description). For both methods, PSI values from each BUA-EEG/ECoG pair were first z-scored and then averaged across different recording pairs. Periods of significant phase locking between BUA-EEG/ECoG were determined using a cluster-based, nonparametric statistical test (described below), which compared the time course of phase locking values aligned to the onset of a EEG/ECoG β bursts (condition A) to that calculated using randomly selected epoch of the same length taken from anywhere in the recording (condition B). The onset of phase locking was defined as the first point when the time course of phase-locking value between BUAβ and EEG/ECoG had a positive derivative before a significant peak (as defined by the cluster-based statistic) in PSI. BUA-EEG/ECoG pairs were included in analysis only if the summed coherence between 15 and 35 Hz was greater than 10% of the total coherence from 1 to 500 Hz.

#### Assessment of modulation of BUA_β_ amplitude.

ECoGs and BUAs were down-sampled to 1,000 Hz and filtered ±5 Hz around the peak ECoG-BUA beta coherence using a second-order Butterworth filter (after which they are referred to as ECoG_β_ and BUA_β_). Instantaneous amplitude in the beta frequency band was derived from the magnitude of Hilbert transform of the ECoG_β_ and BUA_β_ signals. A moving average filter of length 50 ms (box filter) was applied to the instantaneous beta amplitude since any amplitude changes in the beta frequency band would occur at a much slower rate than the average period of the rhythm of interest (i.e., 50 ms). Changes in BUA_β_ amplitude were assessed with respect to the median BUA_β_ amplitude −500 to 0 ms before a reference time point (i.e., onset of a cortical β burst). Significance was determined in the same way as described for PSI, by comparing the amplitude modulation to that of randomly selected data using the cluster-based, nonparametric statistical test for statistical comparison of time series described below.

#### Cluster-based nonparametric statistical test for statistical comparison of time series.

Statistical difference between 2 time series (condition A and condition B) was determined using the ft_timelockstatistics function from the MATLAB toolbox FieldTrip. Briefly, a paired *t* test across subjects was computed to compare condition A and condition B. Neighboring samples where the *t* value exceeded that corresponding to a predefined cluster-forming threshold (*P* < 0.05) were considered as clusters, and *t* values inside these clusters were summed, generating the cluster-level *t* statistic. The procedure was repeated 2,000 times while randomly exchanging labels between the 2 conditions for each subject. The values of summed *t* statistics for the clusters of the original condition assignment were compared with the distribution of the maximal summed *t*-statistic values collated across the 2,000 random reassignments. A cluster would be labeled as significant if its summed *t*-statistic value exceeded 97.5% of the randomized distribution. This would correspond to *P* < 0.025 which we use to correct for the fact that we tested for both positive and negative differences.

##### Burst-triggered averaging of spiking activity.

Spiking activity was converted to a binary time series sampled at 1 kHz, with a 1 at the time of the peak of the each spike. To investigate the phase organization in single units, this binary signal was convolved with a Gaussian filter (reciprocal of SD 25 ms) and averaged across all ECoG β bursts, following realignment to the onset of each ECoG β burst. If a unit was not significantly phase locked to the cortical beta (Rayleigh test, alpha = 0.05), or the maximum burst-triggered spiking activity from a probe was less than 1.96 (z score) (within ±200 ms of burst onset), burst-triggered spiking activity derived from that unit was excluded from further analysis. To differentiate between phase-driven modulation and that due to slower changes in firing rate, the same analysis was performed using ECoG β-burst onsets that were not phase adjusted. (See *SI Appendix*, Fig. S10*A*, for schematic description.)

#### Analysis of phase slips.

Instantaneous phase was derived from the Hilbert transform of either the filtered BUA or the filtered ECoG, and phase alignment was computed and corrected as described above (*Phase locking and PSI*). We then differentiated the unwrapped phase alignment and subsequently calculated the z score for each BUA and ECoG pair. When z-scored difference between adjacent unwrapped phase alignment values was greater than 1.96 or less than −1.96, this sample was labeled as a phase slip. Therefore, phase slips included unwrapped phase alignment values that had either a positive derivative greater than 1.96 (z score) or a negative derivative less than −1.96 (z score). Samples labeled as a phase slip were set to 1, while other samples were set to 0, creating a binary time series. Finally, phase slips derived from each BUA-ECoG pair were averaged across recording pairs for the 25 longest cortical β bursts. To compute the time evolution of phase alignment between a BUA-ECoG pair around a phase slip, we computed the average phase alignment within a 50-ms window (i.e., average in time). The angular difference between the average phase alignment between a BUA-ECoG pair −50 to 0 ms before a phase slip and the average phase alignment 0 to 50 ms after the cortical β-burst onset was calculated. This was compared with the angular difference between the average phase alignment between a BUA-ECoG pair 0 to 50 ms after a phase slip and the average phase alignment 0 to 50 ms after the cortical β-burst onset (Fig. 5*C*).

#### Analysis of BUA frequency stability.

BUA frequency stability was derived by filtering the BUA ± 2.5 Hz around the frequency which was most coherent with the ECoG (f_coherent_) and then computing the Hilbert transform of the filtered BUA to derive instantaneous phase. We unwrapped instantaneous phase derived from the Hilbert transform and differentiated the unwrapped phase. We computed instantaneous frequency by scaling the differentiated unwrapped phase by 1,000/(2π). Using a moving window of width 50 ms, we computed the SD of the instantaneous frequency. The moving window was advanced every 1 ms and was not tapered. SD of the instantaneous BUA frequency was aligned to cortical burst onset and averaged across different bursts. Only BUA recordings, which had the summed ECoG-BUA coherence between 15 and 35 Hz greater than 10% of the total coherence between the 2 sites, were included in the analysis. This procedure was repeated for f_coherent_ − 10 Hz and f_coherent_ + 10 Hz. Time course of the SD of the instantaneous BUA frequency, aligned to burst onset (condition A), was compared with the time course of the instantaneous BUA frequency that was not aligned to burst onset (condition B). The cluster-based nonparametric statistical test, described above, was used to determine when these 2 time courses differed significantly.

#### Cycle-by-cycle phase-locking analysis.

The instantaneous phase and power of the ECoG were separately calculated from the analytic signal obtained via the Hilbert transform of 5-Hz-wide filtered signals (Butterworth Filter, second order) across the beta frequency range, with center frequencies of 17.5 to 32.5 Hz and a 2.5-Hz overlap. The phase-locking strength of each spike train across the whole recording was assessed by computing Rayleigh’s test and the vector length using the ECoG β-phase values in each frequency band at the time of every spike. If phase locking was significant at 1 or more beta frequency ranges (Rayleigh’s test, *P* < 0.05), that spike train was included in cycle-by-cycle analysis using the ECoG_β_ phase leading to highest vector length for that neuron. The same frequency range was then also used to define the ECoG β-burst onset. Once the locking frequency had been selected, the minima of the derivative of the ECoG β phase were used to define the indices of the start and end of each cycle around each burst threshold. The cycle containing phase-adjusted burst threshold (i.e., the time point of the oscillation peak of this cycle) was designated cycle 0. Subsequent analyses were conducted on this cycle and 10 cycles on either side. For the spike train of a given neuron, we then extracted the ECoG β phase at the time of every spike occurring in each cycle across all of the ECoG β bursts for that recording (see *SI Appendix*, Fig. S10*B*, for schematic description). These phase values were then used to compute the vector length and mean angle of locking for each cycle with respect to the ECoG β-burst threshold. For each group of neurons (STN, GP-TI, etc.), a Kruskal–Wallis ANOVA was used to determine whether vector length across neurons was significantly modulated by cycle position with respect to ECoG β-burst threshold (*P* < 0.0125 with Bonferoni correction for number of groups).

If a neuron was significantly modulated by ECoG β-burst cycle, differences between the vector lengths of progressive cycle positions were examined. The first cycle with a significant increase in vector length was defined as that with a significantly higher vector length than any of the preceding cycles, as measured by post hoc Dunn–Sidak tests. Significance on subsequent cycles was assessed relative to the cycle preceding the first significant cycle. For each individual cycle (for 1 neuron), Rayleigh’s test (*P* < 0.05) was used to define whether spikes were significantly phase locked to the ECoG_β_ phase (i.e., the phases had a nonuniform distribution). Across a population of neurons, the results of these tests were used to calculate the number of neurons with significant phase locking for a given cycle and the maximum number of consecutive cycles with significant locking across the 21 cycles for each neuron. For a given neuron, if the spikes in a given cycle were significantly locked, the angle of locking for that cycle was calculated as the circular mean of the phases for all spikes in that cycle. The circular mean of these values (across neurons) for each cycle was used to calculate the mean angle of locking in a given cycle for a group of neurons. The Watson–Williams test (*P* < 0.01) was used to test whether there was a significant difference in the mean angle of locking between consecutive cycles. All circular statistical measures were only calculated if there were >20 spikes/phases for a given neuron or cycle. All circular statistical measures were calculated using the CircStat toolbox for MATLAB (48).

## Supplementary Material

Supplementary File
