# Supplementary material for: Temporal evolution of beta bursts in the parkinsonian cortical and basal ganglia network
Source: Proc Natl Acad Sci U S A. 2019 Jul 24;116(32):16095–104. doi: 10.1073/pnas.1819975116 (PMC6690030; doi:10.1073/pnas.1819975116)
Supplement: Supplementary File [file pnas.1819975116.sapp.pdf]

PNAS
www.pnas.org

Supplementary Information for

Temporal evolution of beta bursts in the parkinsonian cortico-basal ganglia network

Hayriye Cagnan, Nicolas Mallet, Christian K.E. Moll, Alessandro Gulberti, Abbey B. Holt, Manfred Westphal, Christian Gerloff, Andreas K. Engel, Wolfgang Hamel, Peter J. Magill, Peter Brown, Andrew Sharott

Dr Hayriye Cagnan

Email: hayriye.cagnan@ndcn.ox.ac.uk

Dr Andrew Sharott

Email: andrew.sharott@pharm.ox.ac.uk

This PDF file includes:

Supplementary text
Figs. S1 to S15
Tables S1 to S4
References for SI reference citations

www.pnas.org/cgi/doi/10.1073/pnas.1819975116

Supplementary Information Text

Intraoperative recordings

Patient details and clinical scores. Please refer to Sharott et al for preoperative patient selection criteria (1). Patient details and clinical scores, together with medication dosages, are detailed in Table S4.

Surgical procedures for PD patients: Please refer to Sharott et al for surgical details (1). The surgical target was determined using a combination of an MRI-compatible frame (Stryker Leibinger, Freiburg, Germany) and individual computerized tomography scan with T1 and T2 weighted MRI. A commercially available algorithm was used to fuse different neuroimaging modalities (iPlan, BrainLAB Inc., Westchester, IL, USA). Fused images were used to define the STN/nigra complex, the anterior/posterior commissure, and blood vessels. The surgical target (i.e. STN) was defined as 11-13 mm lateral to the midline, 1-3 mm inferior and 1-3 mm posterior to the mid-commissural point on both hemispheres. Electrode trajectories used for microelectrode recordings aimed to avoid sulci, blood vessels and ventricles.

Parkinsonian Rats

Adult male Sprague Dawley rats (Charles River) were used for all experimental recordings. All experimental work adhered to Society for Neuroscience Policies on the Use of Animals in Neuroscience Research and were in agreement with the Animals (Scientific Procedures) Act, 1986 (United Kingdom). Animals were housed in a temperature-controlled environment with a 24hr light/dark cycle and *ad libitum* access to food and water.

6-Hydroxydopamine lesions of midbrain dopamine neurons. Please refer to the following papers for details on 6-hydroxydopamine (6-OHDA) lesions (2-5). All surgical procedures were performed during the day in a dedicated surgery room. 6-OHDA lesions took place under anesthesia. Anesthesia levels were assessed regularly by testing reflexes to a cutaneous pinch. In order to lesion midbrain dopamine neurons, 1 µl of 6-OHDA solution was injected 4.1 mm posterior and 1.2–1.4 mm lateral of Bregma, and 7.9 mm ventral to the dura (Paxinos *et al.*, 2007). Following surgery, animals were closely monitored and assessed for recovery using several metrics. Only animals that were judged to have recovered sufficiently were assessed for the severity of lesion and used for electrophysiological recordings. Lesion efficacy was assessed 15 days after 6-OHDA injection. The lesion was classified as effective when animals performed ≥ 80 contraversive rotations in 20 min following apomorphine administration (0.05 mg/kg, s.c.; Sigma) (2).

Silicon Probe recordings. Electrophysiological recordings were performed 21–39 days after 6-Hydroxydopamine lesions of midbrain dopamine neurons under anesthesia. Details on silicon probe recordings have been previously described (5, 6). High impedance unity-gain operational amplifiers were used to record monopolar signals from each probe contact (Advanced LinCMOS; Texas Instruments). The epidural electrocorticogram (ECoG) was recorded with a 1 mm-diameter screw above the frontal cortex (4.2 mm rostral and 2.0 mm

lateral of Bregma; (7), ipsilateral to basal ganglia recordings. An equivalent screw above the contralateral cerebellum was used as reference. Signals (ECoG and probe) were each sampled at 16.7 kHz using a Power1401 amplifier and Spike2 software from Cambridge Electronic Design Limited. Anesthesia levels were assessed by examination of the ECoG and by testing reflexes to a cutaneous pinch. Following electrophysiological recordings, animals were euthanized. A fixative was used for transcardial perfusion (5, 6).

The probe signals were bandpass filtered between 500 and 6000 Hz. Single-unit activity was extracted according to the following criteria: 1) signal/noise ratio of >2.5 and 2) spike sorting using methods such as template matching, principal component analysis, and supervised clustering (Spike2 Cambridge Electronic Design Limited). Units were classified as single units if a distinct refractory period in the inter-spike interval histograms could be identified. Spiking activity recorded from the GPe was assigned to Type A (TA) or Type I (TI) populations based on whether they preferentially discharged on the positive "active" or negative "inactive" component of the cortical 1Hz oscillation during the slow wave brain state using activation histograms (5), visual inspection of the raw data and the lag of the central peak in the spike triggered average of the cortical ECoG. Spike trains that did not fit unambiguously into the TA or TI group were not considered further in this study.

***Simulation of burst triggered average analysis*.** One second simulations were run to determine how beta burst lengths and frequencies affect the burst-triggered average analysis used in Fig. 4. Beta bursts were generated by multiplying a sine wave of a specified frequency by a Gaussian, whose width and height were randomly selected from a specified range. This created a burst whose peak height occurred at the midpoint. Burst duration could be altered by manipulating the width of the Gaussian, while frequency could be altered via the sine wave. 1000 bursts were generated for each simulation and each was realigned to the start of the burst, detected using an amplitude threshold of 1e-3. Next, a simulated spike train was generated based on a firing rate and firing probability, sampled at 1000 Hz. The amplitude of the beta burst was used to represent spiking probability, with 1 being 100%. Multiplying the probability vector by the firing rate resulted in noisy spiking that was modulated by phase. In the simulations, we generate a spike train with the assumptions that spiking occurs preferentially at a certain phase of the burst, and that this phase is stable across bursts. These conditions are sufficient to generate oscillatory activity when averaging across bursts. Overall, results show that while such oscillatory modulation, detected by averaging spiking across beta bursts, depends on the distribution of lengths and frequencies of bursts, it can be seen when those conditions are matched to those of the recordings. As variable length and frequency across bursts dilute the emergence of an oscillation in the averaged spiking activity, significant modulation of the burst triggered average indicates a robust modulation from phase-locking.

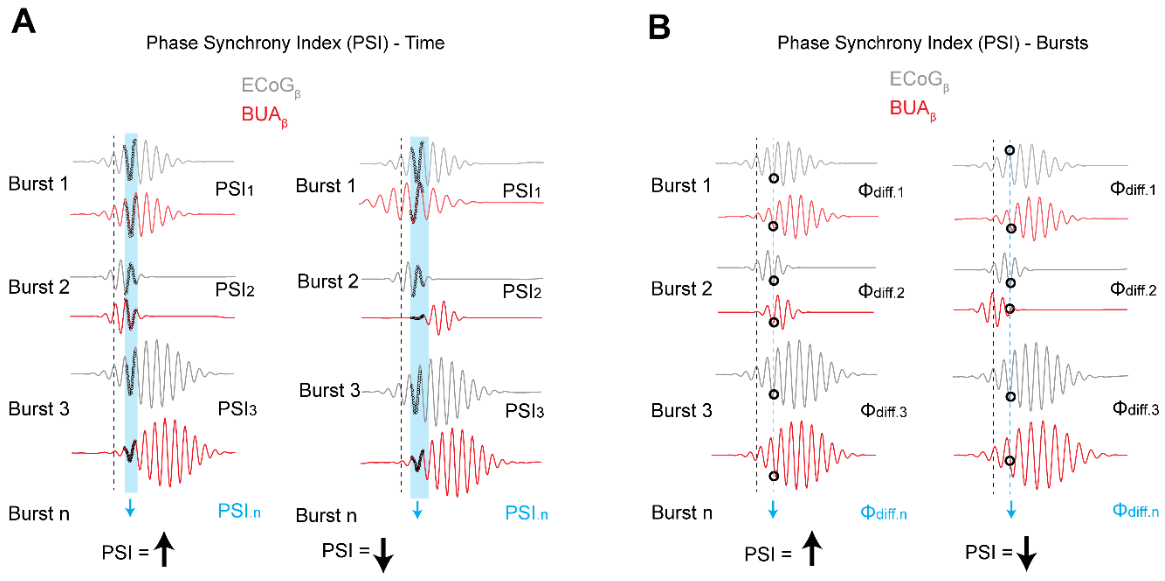

Figure S1. Schematic examples of methods of analyzing phase-locking between the EEG/ECoG and background unit activity (BUA). To analyze phase-locking between ECoG and BUA, both signals were first filtered in the beta frequency band, and the phase of these signals was extracted using the Hilbert transform (denoted by $ECoG_β$ and $BUA_β$, this example would be the same for the $EEG_β$ in the human data). These filtered signals are represented by idealized sine waves in both plots. Pairs of $ECoG_β$ and $BUA_β$ signals were then analyzed using two variations of the phase synchrony index (PSI), which essentially measures the consistency (vector length) of the difference between series of phases (for details of calculation, see methods). The figure is a purely schematic representation of the factors that contribute to different outcomes of PSI analyses. **A) PSI calculated over time.** The PSI is calculated from the all the data points in a 50ms window (blue bar, data points within represented by open circles) at given time in relation to the burst threshold (black dotted line) for each burst individually. These PSI values for each burst are then averaged to give a mean PSI value for each time window. High mean values thus result from high synchronization in that time window in each individual burst, irrespective of whether the phase difference changes (e.g. due to changes in frequency and/or polarity in both signals) between bursts (left). Low mean values can be the result of differences in frequency and/or alignment of periods of high signal-to-noise between signals in each individual burst. **B) PSI calculated over bursts.** The phase difference is calculated at one time point for each burst (black open circles). The vector length of those phase differences, for each point across bursts (corresponding to the point plotted on the x-axis), is used to calculate the PSI. Inconsistency of phase differences, which can be due to the two signals having different frequencies and/or polarities *across* bursts, results in a low PSI for that time point (right). The measure therefore indicates the *consistency* of phase difference between two signals at a given time point in relation to the burst threshold, across different bursts. If both across time and across burst measures increase at a given point in time/window (in relation to the burst threshold), synchronization is increased in individual bursts, and the phase relationship (conditions of synchronization) is consistent.

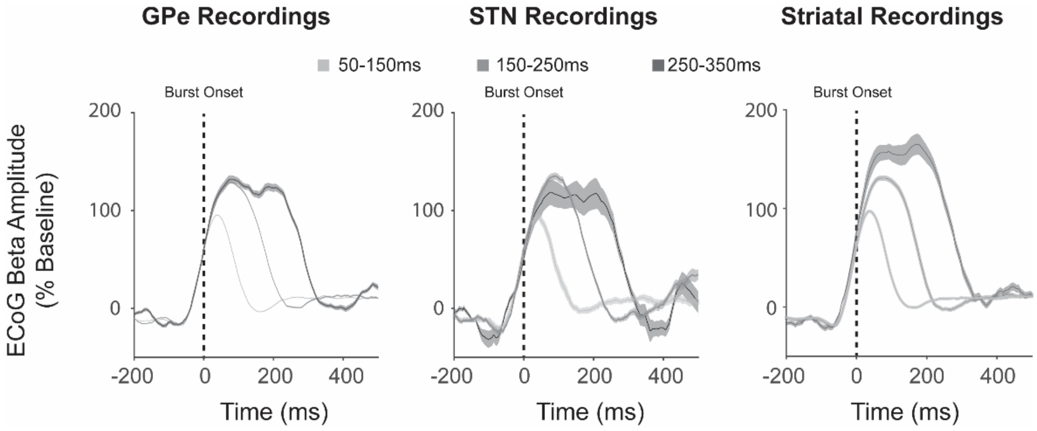

Fig. S2. ECoG beta bursts of during BG BUA recordings. The normalized amplitude envelope of the beta-filtered ECoG ($ECoG_{\beta}$) was aligned to the cortical β-burst onset, defined according to the crossing of the 75th percentile of the amplitude for the entire recording. The onset of a cortical β-burst is indicated with a dashed line at time 0 ms. The resulting β-bursts were grouped into three durations (color key). The three panels show the cortical β-bursts for the corresponding basal ganglia structures with which they were simultaneously recorded (aligned to the burst onset, defined according to the 75th percentile crossing).

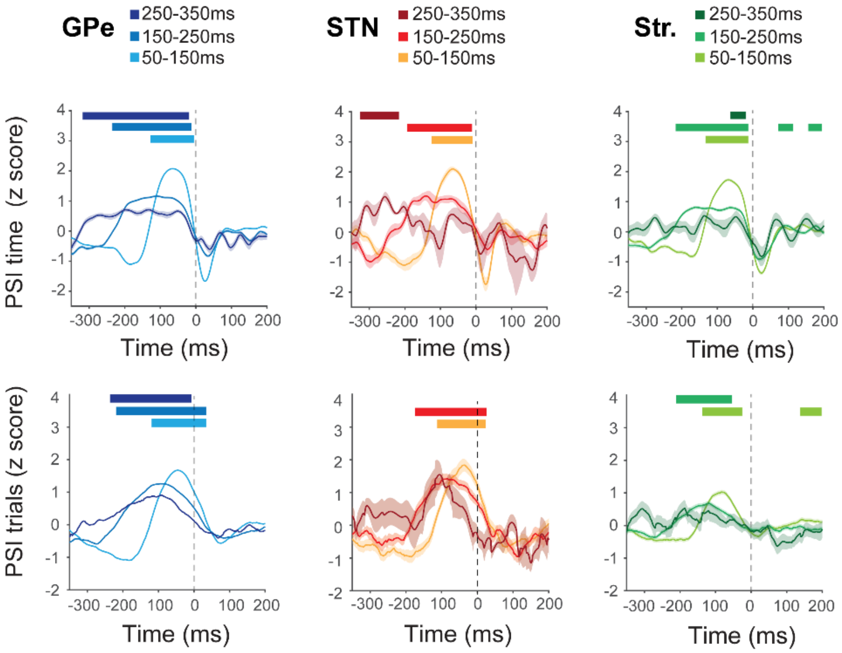

Fig. S3. Cortico-basal ganglia phase locking at the offset of ECoG β-bursts. Top row, phase synchrony index (PSI), computed in time, between $ECoG_{\beta}$ and each basal ganglia BUA_{β}, aligned to the offset of the ECoG β-burst. Bottom row, PSI, computed across bursts, between ECoG and each basal ganglia BUA, aligned to the offset of the ECoG β-burst. In each case, bursts have been separated into 3 different durations (see color key). Significance for each burst length was determined using a cluster-based analysis (see Methods) which tested the difference in the modulation of PSI over time in the burst-aligned data to that of randomly selected data that had no relationship to burst onset. Significant increases with respect to baseline are indicated with horizontal bars, color matched to the burst duration. (Shaded regions indicate the standard error of the mean across all recordings made from a given structure)

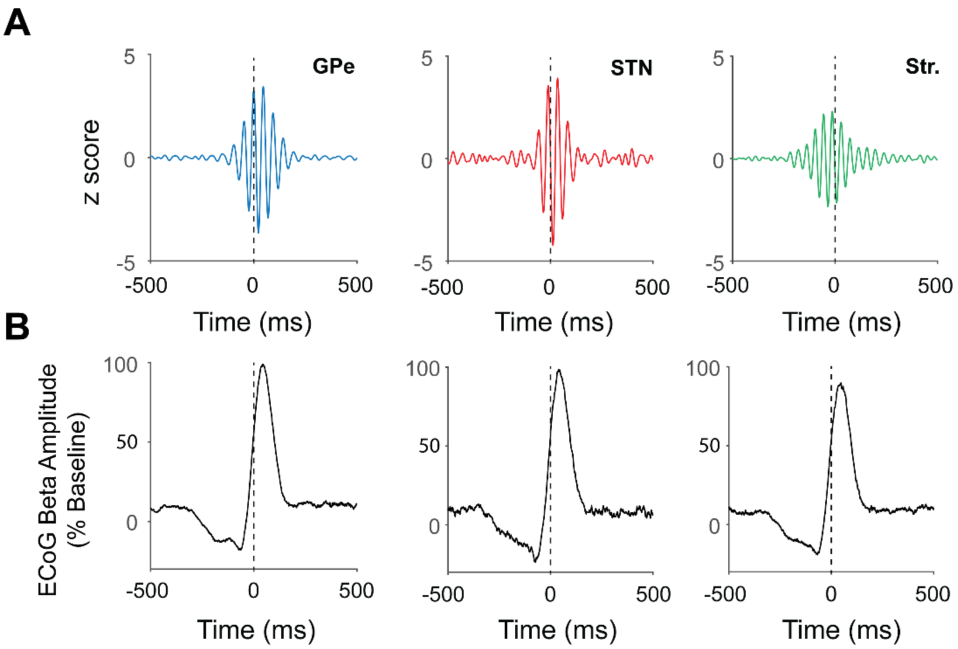

Fig S4. Consistent alignment of unfiltered BUA to ECoG β-phase before and during ECoG β-bursts. A, The GPe, STN and striatal (Str.) unfiltered BUA was averaged around the nearest peak of the ECoG$_{\beta}$ phase to the amplitude-based burst onset, such that zero-time always had the same ECoG$_{\beta}$ phase (colored lines, left y-axis). Traces show the mean across recordings. In each structure, peaks and troughs in the amplitude of the unfiltered BUA signal, with a period of around 50ms, arose before and after the burst threshold indicating alignment with the cortical oscillation. **B,** The same calculation was performed for ECoG$_{\beta}$ amplitude envelope (gray lines, right y-axis) for the coincident ECoG recordings made with those of each basal ganglia population.

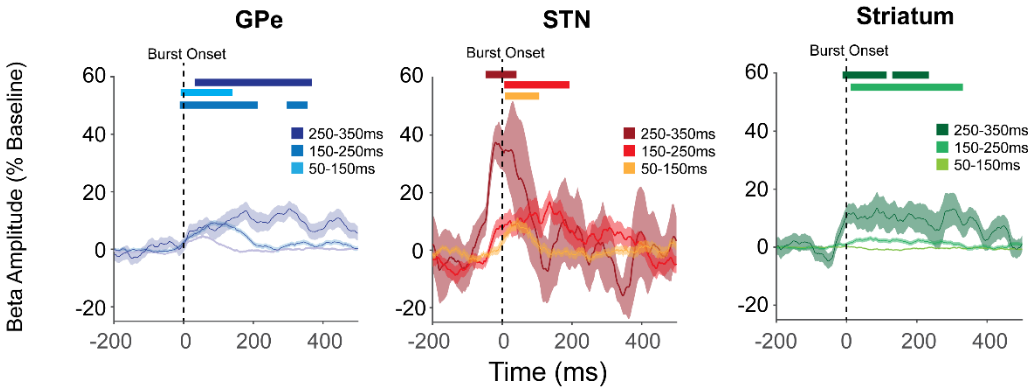

Figure S5. GPe, STN and striatum BUA$_\beta$ amplitude exhibits enhanced oscillatory activity in the beta frequency band during ECoG β-bursts. The amplitude envelope of the beta-filtered BUA activity from the basal ganglia aligned to the ECoG β-burst onset, defined according to the 75th percentile crossing. Significant increases with respect to baseline are indicated with horizontal bars, color matched to the burst duration. Significance for each burst length was determined using a cluster-based analysis (see Methods) which tested the difference of the modulation of BUA$_\beta$ amplitude over time in the real data to that in randomly selected data with no relationship to burst onset. Significant increases with respect to baseline are indicated with horizontal bars, color matched to the burst duration. (Shaded regions indicate the standard error of the mean across all recordings made from a given structure)

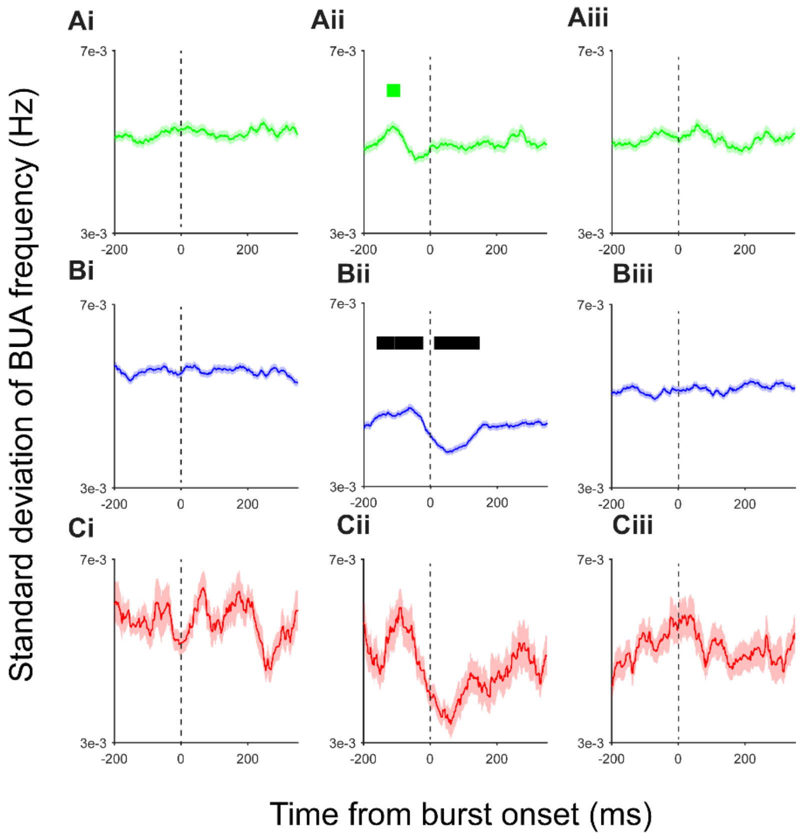

Figure S6. Frequency stability in basal ganglia BUAs. BUA frequency stability was derived by filtering the BUA ±2.5 Hz around the beta frequency which was most coherent with the ECoG ($f_{coherent}$) and the neighboring frequency bands (see methods for full details of calculation). Standard deviation of the instantaneous BUA frequency for $f_{coherent}$ – 10 Hz (left), $f_{coherent}$ (middle) , $f_{coherent}$ + 10 Hz (right) while rows indicate striatal (green), pallidal (blue), subthalamic (red) BUA respectively. 0 ms indicates cortical burst onset while y-axis shows standard deviation of the instantaneous BUA frequency. We observed that the standard deviation of the instantaneous BUA frequency momentarily increased prior to burst onset followed by a reduction after cortical burst onset for BUA frequencies ±2.5 Hz around $f_{coherent}$. For pallidal BUA, both these time periods were significantly different from the standard deviation of the instantaneous BUA frequency, which was not aligned to a cortical burst onset. For striatal BUA, only the early increase in the standard deviation of the instantaneous BUA frequency was significant, while for subthalamic BUA neither the early increase nor the post cortical burst onset reduction in the standard deviation of the instantaneous BUA frequency was significant. Statistical difference between the time courses of the standard deviation of the instantaneous BUA frequency aligned and not aligned to cortical burst onset was computed using the cluster-based test outlined in the methods section.

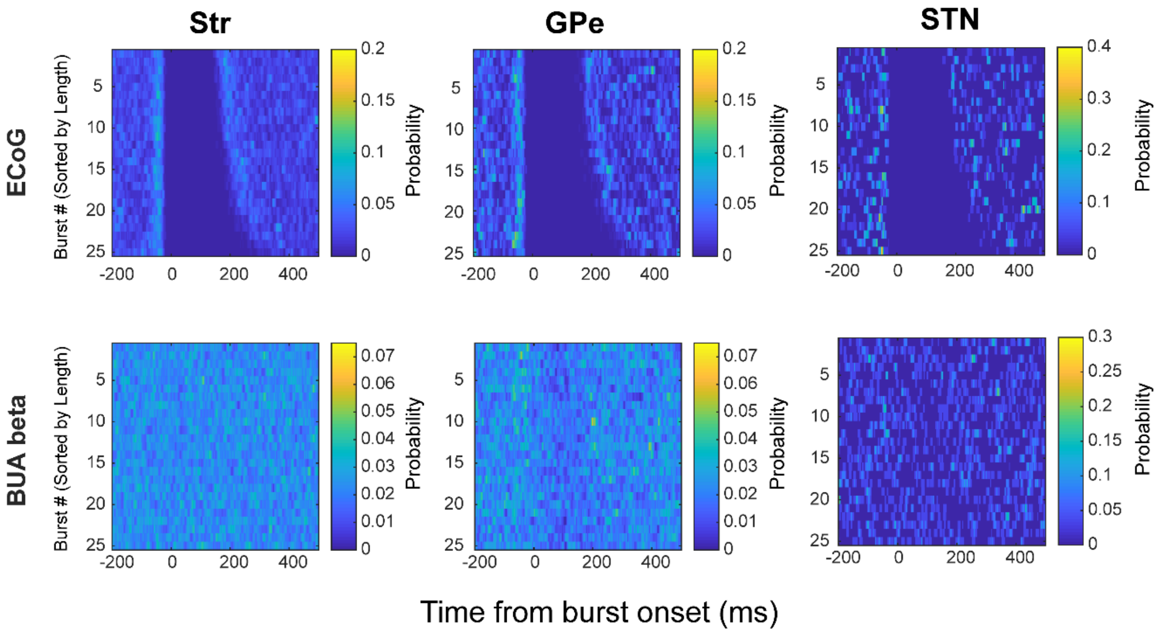

Figure S7. Phase-slips occurred in ECoG$_\beta$ and basal ganglia BUA$_\beta$ recordings around β-burst onset. Phase slips could be observed in unwrapped Hilbert phase of both ECoG$_\beta$ and BUA$_\beta$ signals during the 25 longest ECoG β-bursts in each recording. The average probability of an ECoG$_\beta$ phase slip occuring in the 200ms prior to β-burst onset was greater than the average probability of an ECoG$_\beta$ phase slip occurring in the following 200ms (panels A-C $p<0.001$). However, the average probability of a BUA$_\beta$ phase slip in the 200ms prior to β-burst onset was greater than the average probability of a BUA$_\beta$ phase slip occurring in the following 200ms for the STN and GPe, but not for the Str (paired t tests, panel D $p=0.0943$; panel E $p<0.001$; panel F $p=0.0196$).

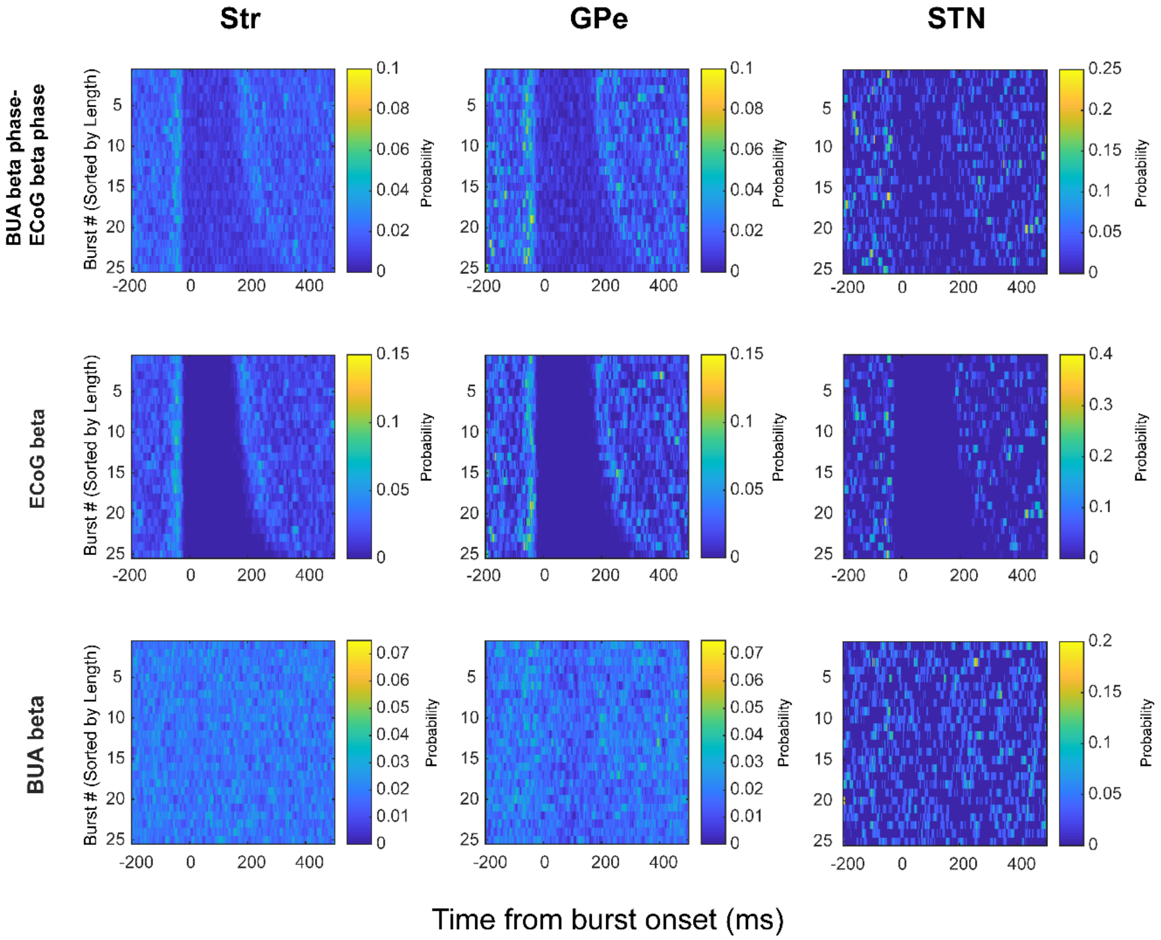

Figure S8. Phase-slips within and between ECoGβ or BUAβ are still detected after removal of epochs of complete oscillatory break-down. Average phase slips observed during the 25 longest ECoG β-bursts after points corresponding to a negative frequency either in ECoGβ or BUAβ were removed. This additional criterion did not impact the results shown in Fig 3A. The average probability of a phase slip (derived from the difference between the Hilbert phase angles of ECoGβ and BUAβ pairs) in the 200ms prior to β-burst onset was greater than the average probability of a phase slip occurring in the following 200ms (panels A-C, $p<0.001$). As highlighted in Fig. S7, phase slips could be observed in both ECoGβ and BUAβ. The average probability of an ECoGβ phase slip in the 200ms prior to β-burst onset was greater than the average probability of an ECoGβ phase slip occurring in the following 200ms (panels D-F, paired t test: $p<0.001$). However, the average probability of a BUAβ phase slip in the 200ms prior to β-burst onset was greater than the average probability of a BUAβ phase slip occurring in the following 200ms only for the STN and GPe but not for the Str (paired t tests, panel G $p=0.46$; panel H $p<0.001$; panel I $p=0.0484$).

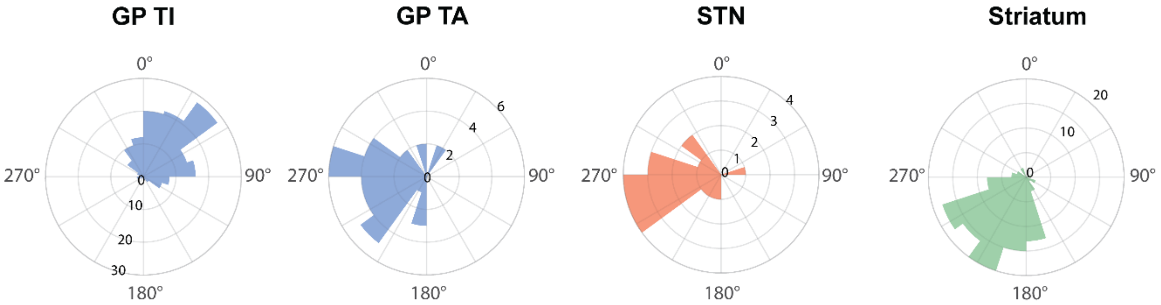

Figure S9. Mean phase locking angles of basal ganglia neurons on ECoG beta oscillations. Histograms of the mean angles of phase locking for the action potentials of single units from the different basal ganglia neuronal populations.

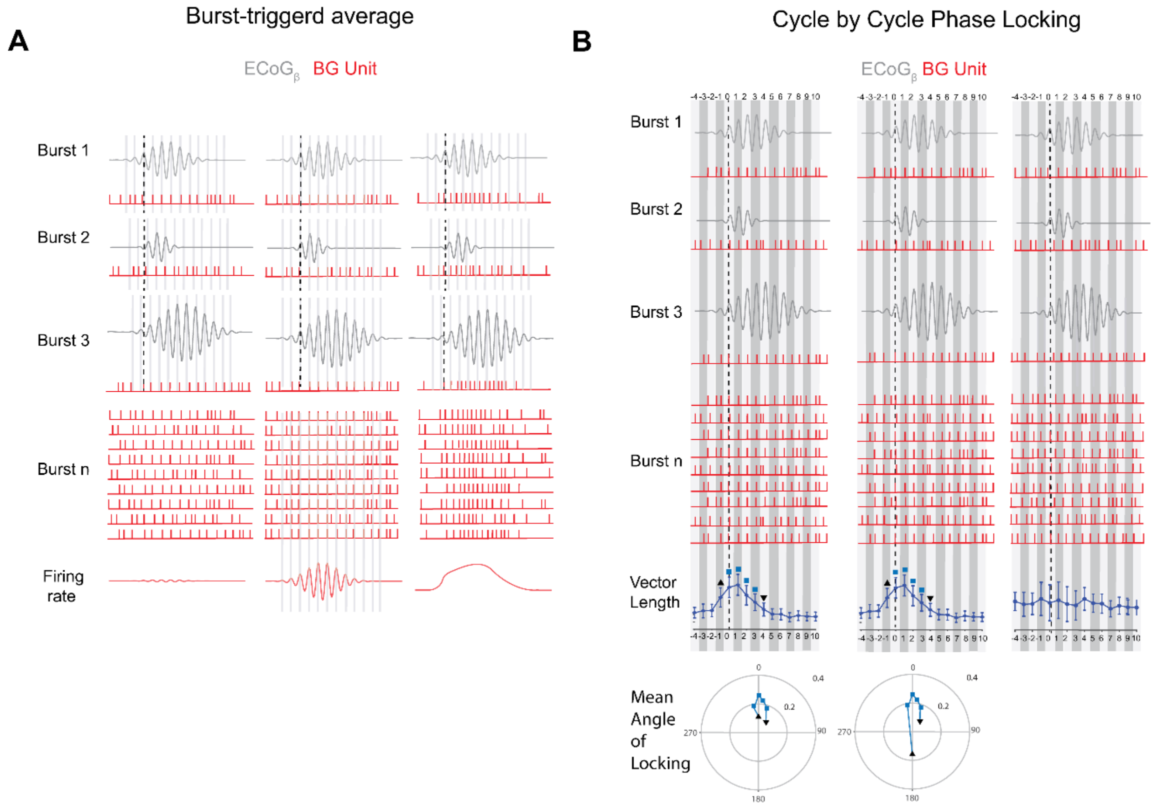

Figure S10. Schematic examples of methods for analyzing phase-locking between the ECoG and single unit activity. To analyze phase-locking between the ECoG and action potentials recorded from single units, the ECoG was filtered in the beta frequency band (then denoted by $ECoG_{\beta}$) and the phase extracted using the Hilbert Transform. The filtered ECoG signals at the time of a β-burst are represented by the gray sine waves. The timing of action potentials are represented by the red vertical lines. These signals were primarily used in two related, but complementary ways. The figure is a purely schematic representation of the factors that could contribute to different outcomes of our analyses of unit activity. **A)** Burst-triggered average. Spike counts were triggered by the burst onset (the time point cortical beta amplitude exceeded the 75th percentile for the whole recording). **Left,** spikes can be phase-locked to the same phase of the ECoG for during the burst, but this does not sum up across trials if the burst-trigger is not aligned to the same phase. **Middle,** using the same ECoG/unit time series, the burst-trigger is adjusted to the nearest oscillation peak. Because the phase is constant at a given point in time, the spikes align and sum across the bursts giving an oscillatory modulation of firing rate. **Right,** if spikes consistently increase in rate over a slower timescale than the oscillation frequency within the burst period, they will sum across bursts as a broad, non-oscillatory modulation of firing rate, irrespective of whether the burst-trigger is phase-aligned. For the analysis in Fig. 4, binary action potential signals were smoothed using a Gaussian filter, but the principles of the technique are identical. **B)** Cycle-by-cycle phase locking. Each ECoG β-burst was divided into oscillation cycles, represented by alternating light and dark bars. The phases at the times of all action potentials in a single cycle were extracted and used to calculate circular statistical measures for each individual cycle across bursts. Raleigh tests

were used to define whether the distribution of action potential phases was non-uniformly distributed, indicating significant phase locking. Vector length was used to measure the consistency of the phase of spikes in each cycle. The mean phase angle was used to describe the preferred/mean phase of spikes on a given cycle. These values are independent of frequency/cycle length both across time and across bursts, as the phase value for a given action potential is bound within 0 and 360 degrees irrespective of variations in cycle length. **Left,** Spikes are aligned to the peak of the ECoG oscillation from one cycle before (black triangle) that containing the burst threshold (black dotted line) to four cycles after (inverted triangle). The circular plot shows the mean phase angles (position around the circle) of the spikes in the cycles with increased vector length (distance from the center). In this example, spikes are always locked close to the oscillation peak (0°), shown on the circular plot below. **Middle,** as in the left plots, except that the spikes in the first cycle with increased vector length (black triangle) lock at the oscillation trough, as highlighted in the circular plot below. **Right,** if spikes are not consistently aligned to any particular phase in any cycle, the vector length plot is flat and mean phase angles cannot be considered.

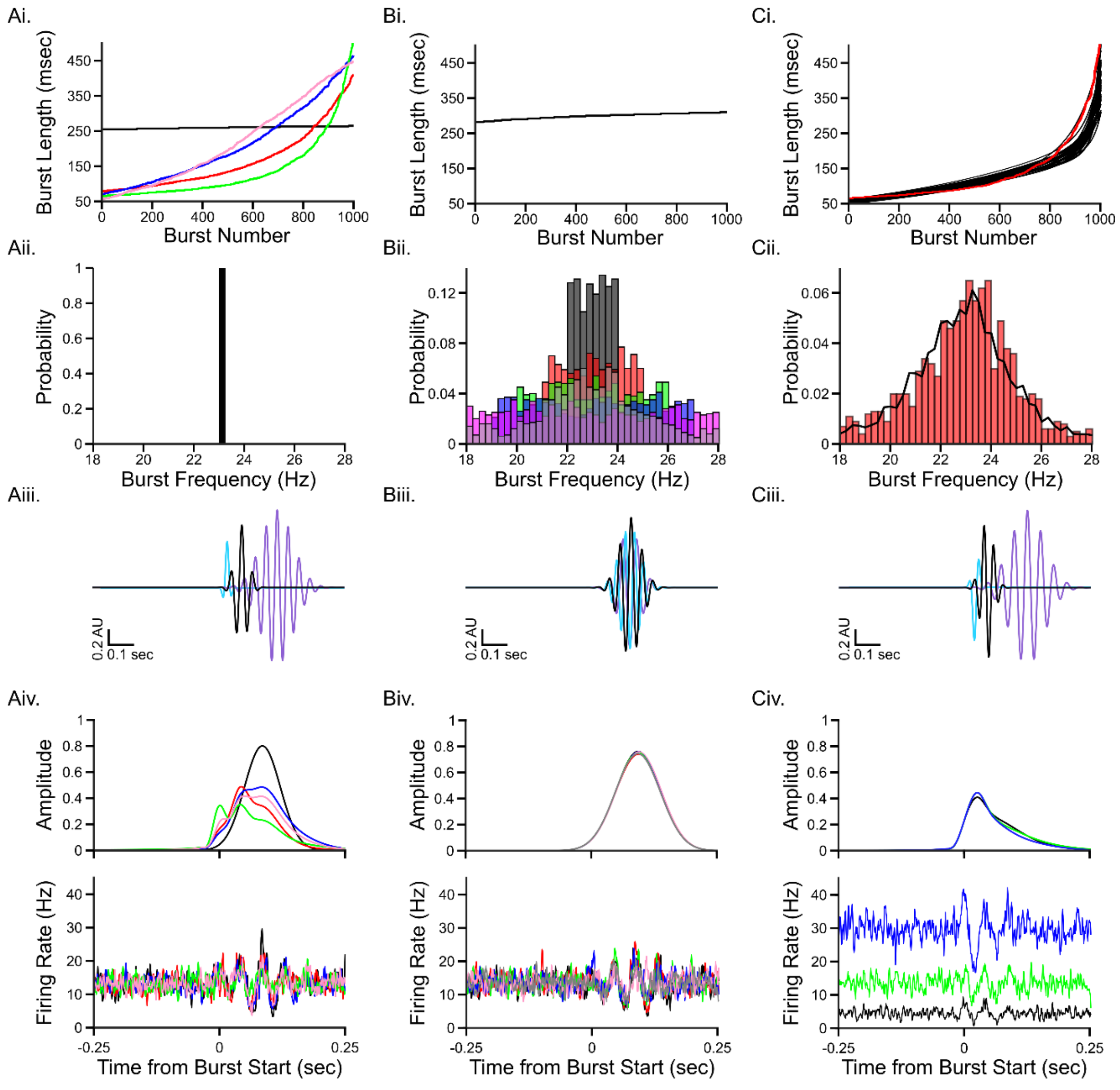

Figure S11. Oscillatory unit activity could be seen in simulations despite varying the length and frequency of β-bursts. In simulations **A.** β-burst lengths were varied, while the frequency was held at 23 Hz; **B.** β-burst frequency was varied while lengths were held constant; and **C.** length and frequency distributions of β-bursts were set to mimic what was seen in recordings. **Ai, Bi, Ci.** The various lengths of β-bursts can be seen. In Ci, the distribution of lengths in simulations (red) was set to represent lengths seen across recordings (black). **Aii, Bii, Cii**. Histograms show the frequency distribution of β-bursts. In Cii, the distribution of frequencies in simulations (red) was set to match the average seen across real recordings (black). **Aiii, Biii, Ciii.** Examples of β-bursts aligned to the onset for each condition**. Aiv Biv, Civ.** Top: average amplitude of β-bursts. Bottom: Burst-triggered average of simulated units. In Civ results can be seen for three firing rates representing GPe (green), STN (blue), and striatum (black). The distribution of both burst length and frequency affect the oscillatory behavior of the simulated unit, however robust modulations in the triggered-averages were reproduced at different firing rates (approximating the main

neuronal populations studied) using variances of these parameters that was matched to that of the real data.

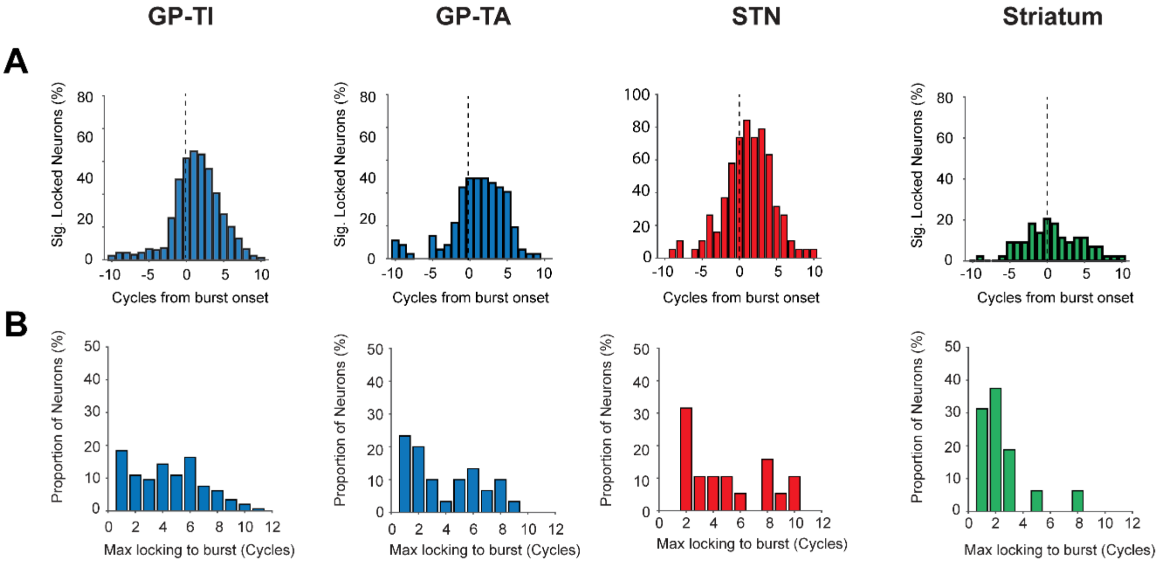

Figure. S12. Cycle-to-cycle characteristics of BG neuron phase-locking during ECoG β-bursts. A) Proportion of neurons of each type that were significantly ECoG beta oscillation in each cycle with respect to the β-burst onset (dotted line **B)** Histograms of the maximum number of consecutive cycles in the burst period to which each neuron of a given type was significantly phase locked. Significance for a given cycle in both cases was defined using Rayleigh's Test ($p < 0.05$).

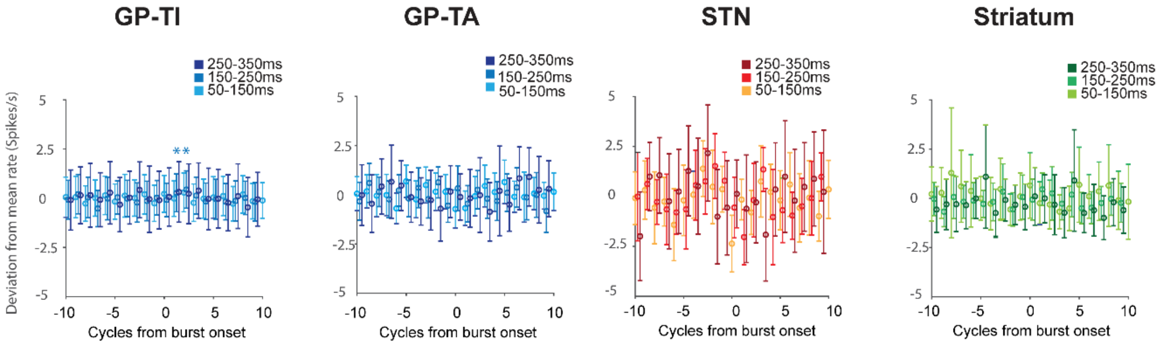

Figure S13. Firing rate is rarely modulated by ECoG bursts. Mean firing rate ± 1SD for each cycle in relation to the ECoG β-burst threshold for each neuron type, separated by burst duration (color code). Only GP-TI neurons during medium (150-250ms) bursts displayed any modulation in firing rate (Kruskal Wallis Anova, $p = 0.0114$), with a significant difference for the two cycles after burst onset (asterisks). The increases were <0.5 spikes/s above any previous cycle. Otherwise, there were no significant differences in firing rate between cycles for any burst duration for any neuron type (Kruskal Wallace Anova with post-hoc Dunn-Sidak tests to test if values were significantly different to any previous cycle).

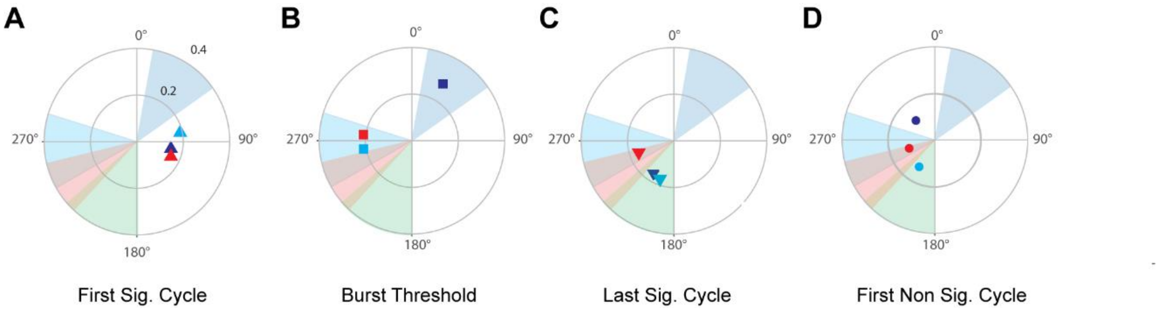

Figure. S14. Preferred phases of the spikes of basal ganglia single unit activities in relation to the temporal evolution of the ECoG β-burst. The phase angles and vector lengths of spikes of GP-TI (dark blue), GP-TA (light blue) and STN (red) at different stages ECoG β-burst temporal evolution. All data points are taken directly from Fig.5 (and symbols are matched to that figure), but plotted together here to aid comparison of the different neuron-types at the key points in the ECoG β-burst.

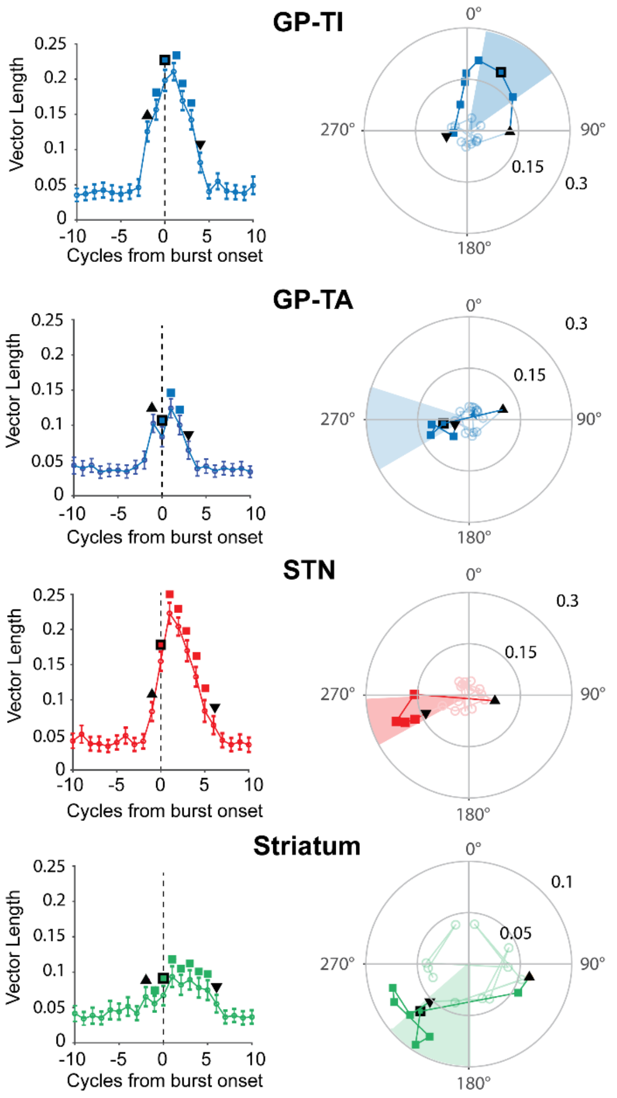

Figure S15. Phase locking strength and conditions of basal ganglia single unit activities during ECoG β-bursts calculated with equal numbers of spikes. Left, Cycle by cycle vector length was calculated for each population, as in Figure 5, but using a resampled data constructed from randomly selected phases from any spike recorded across all neurons of one type for a given cycle. Using this method, phases used to calculate vector length are not always derived from the action potentials of the same neuron, but that the same amount of spike/phase values can used for each population, irrespective of the firing rate of the neurons or the number recorded, which were much lower in striatum and STN respectively This approach led to a similar pattern of phase-locking strength in relation to the ECoG burst onset as the original analysis (Fig. 5), but led to statistical significance for striatal phase-locking during cycles -2 to 6, which was not seen using the original data set. The permutation-based analysis gave identical results to the original analysis for GPe and STN in terms of the first cycle where significant locking occurred. **Right,** phase trajectories for resampled data, as calculated in Figure 5. Trajectories for GP-TI, GP-TA and STN neurons are similar to the original data. The trajectory for striatal neurons shows that, using

the same number of spikes as the other neuron types, the preferred angle of locking moves almost 180 degrees from the pre-burst threshold to the post-burst threshold cycles, after which spikes lock at or close to the mean beta phase. Note the different vector length scale for striatal neurons. A, significant changes in vector length were defined by Kruskal Wallis ANOVAs followed by post-hoc Dunn-Sidek tests for each neuron type (see Methods). A and B, triangle = first significant increase in vector length, black square = cycle containing burst threshold, colored squares = cycles with significantly increased vector length, inverted triangle = last cycle with significantly increased vector length, faded circles = cycles with non-significant vector length.

**PSI time**	**50-150 ms**	**150-250 ms**	**250-350 ms**
**GPe (n=366)**	-103 ms	-60 ms	-25 ms
**STN (n=22)**	-99 ms	-63 ms	-77 ms
**Str (n=209)**	-97 ms	-42 ms	-89 ms

Table S1. Latencies of onset of increased PSI calculated in time in relation to the ECoG burst threshold.

PSI bursts	50-150 vs. 150-250 ms	50-150 vs. 250-350 ms	150-250 vs. 250-350 ms
**GPe (n=366)**	-95 ms	-68 ms	-35 ms
**STN (n=22)**	-89 ms	-35 ms	-16 ms
**Str (n=209)**	-89 ms	-84 ms	-62 ms

Table S2. Latencies of onset of increased PSI calculated across bursts in relation to the ECoG burst threshold.

**Mean Firing Rate**	
**GP-TI (n=179)**	13 ± 7 spikes/sec
**GP-TA (n=40)**	16 ± 10 spikes/sec
**STN (n=18)**	32 ± 14 spikes/sec
**Str (n=104)**	5 ± 6 spikes/sec

Table S3. Firing rates of neurons used for burst-triggered firing rate and pattern analysis.

Case	Age (Yrs) and Sex	Disease Duration (Yrs)	Motor UPDRS OFF	Motor UPDRS ON	Medication pre-operation	Hoehn/ Yahr Score	Dominant side	Major Symptoms	Hemispheres analyzed
1	61F	25	50	30	Levodopa 700 mg<br>Carbidopa 150 mg<br>Entacapone 1000 mg<br>Benserazide 25 mg<br>Pramipexol 0.26 mg	5	Left	Tremor,<br>Akinesia/rigidity<br>Fluctuations	Right
2	70F	8	38	21	Ropinirole 8mg<br>Alpha-dihydroergocryptine 40 mg<br>Amantadine 600 mg<br>Levodopa 250mg<br>Carbidopa 150mg	3	Left	Bradykinesia<br>Fluctuations<br>Dyskinesia	Both
3	67M	25	55	19	Levodopa 1250 mg<br>Entacapone 1400 mg<br>Carbidopa 312.5mg<br>Rotigotine 6 mg<br>Amatadine 150mg	3	Right	Equivalence<br>Fluctuations<br>Dyskinesia	Both
4	64M	15	53	39	Amantadine 300 mg<br>Levodopa 550 mg<br>Ropinirole 20 mg<br>Entacapone 900 mg<br>Carbidopa 112.5 mg<br>Benserazide 25 mg	4	Left	Akinesia/rigidity<br>Camptocormia	Left
5	69F	9	21.5	7	Levodopa 450 mg<br>Lisuride 0.9 mg<br>Rotigotine 4 mg<br>Amantadine 300 mg	3	Right	Equivalence type<br>Fluctuations<br>Dyskinesia	Both

6	66M	11	28	14	Amantadine 150 mg<br>Levodopa 1450 mg<br>Tolcapone 300mg<br>Carbidopa 312.5<br>Benserazide 50mg	4	Right	Akinesia/rigidity<br>Fluctuations	Both
7	70F	26	45	24	Entacapon 1600 mg<br>Amantadin 300 mg<br>Pramipexol 0.8755 mg<br>Madopar 125 T (Levodopa 100 mg + Benserazid 25 mg) 8x0.5<br>Nacom 100 retard (Levodopa 100 mg + Carbidopa 25 mg) 1x1<br>Selegilin 5 mg	4	Left	Tremor,<br>Akinesia/rigidity	Left

Table S4. Patient Details and Clinical Scores

References

1. Sharott A, *et al.* (2018) Spatio-temporal dynamics of cortical drive to human subthalamic nucleus neurons in Parkinson's disease. *Neurobiol Dis* 112:49-62.
2. Abdi A, *et al.* (2015) Prototypic and arkypallidal neurons in the dopamine-intact external globus pallidus. *J Neurosci* 35(17):6667-6688.
3. Mallet N, Ballion B, Le Moine C, & Gonon F (2006) Cortical inputs and GABA interneurons imbalance projection neurons in the striatum of parkinsonian rats. *J Neurosci* 26(14):3875-3884.
4. Mallet N, *et al.* (2008) Parkinsonian beta oscillations in the external globus pallidus and their relationship with subthalamic nucleus activity. *J Neurosci* 28(52):14245-14258.
5. Mallet N, *et al.* (2008) Disrupted dopamine transmission and the emergence of exaggerated beta oscillations in subthalamic nucleus and cerebral cortex. *J Neurosci* 28(18):4795-4806.
6. Magill PJ, Sharott A, Bolam JP, & Brown P (2006) Delayed synchronization of activity in cortex and subthalamic nucleus following cortical stimulation in the rat. *J Physiol* 574(Pt 3):929-946.
7. Paxinos G & Watson C (2007) *The Rat Brain in Stereotaxic Coordinates* (Academic (Elsevier), Amsterdam) 6th Ed.
